# Metabolomic Profile Reveals That Ceramide Metabolic Disturbance Plays an Important Role in Thoracic Aortic Dissection

**DOI:** 10.3389/fcvm.2022.826861

**Published:** 2022-02-08

**Authors:** Hang Yang, Fangfang Yang, Mingyao Luo, Qianlong Chen, Xuanyu Liu, Yinhui Zhang, Guoyan Zhu, Wen Chen, Tianjiao Li, Chang Shu, Zhou Zhou

**Affiliations:** ^1^State Key Laboratory of Cardiovascular Disease, Beijing Key Laboratory for Molecular Diagnostics of Cardiovascular Diseases, Diagnostic Laboratory Service, Fuwai Hospital, National Center for Cardiovascular Diseases, Chinese Academy of Medical Sciences and Peking Union Medical College, Beijing, China; ^2^State Key Laboratory of Cardiovascular Disease, Center of Vascular Surgery, Fuwai Hospital, National Center for Cardiovascular Diseases, Chinese Academy of Medical Sciences and Peking Union Medical College, Beijing, China

**Keywords:** thoracic aortic dissection, metabolomics, ceramide, macrophage, NLRP3

## Abstract

**Aims:**

Thoracic aortic dissection (TAD) is a life-threatening disease with no effective drug therapy thus far. New therapeutic targets and indications for timely surgical intervention are urgently needed. Our aim is to investigate new pathological mechanisms and potential biomarkers of TAD through global metabolomic profiling of aortic aneurysm and dissection patients.

**Methods and Results:**

We performed untargeted metabolomics to determine plasma metabolite concentrations in an aortic disease cohort, including 70 thoracic aortic aneurysm (TAA) and 70 TAD patients, as well as 70 healthy controls. Comparative analysis revealed that sphingolipid, especially its core metabolite C18-ceramide, was significantly distinguished in TAD patients but not in TAA patients, which was confirmed by subsequent quantitative analysis of C18-ceramide in a validation cohort. By analyzing our existing multiomics data in aortic tissue in a murine TAD model and TAD patients, we found that an enhanced ceramide *de novo* synthesis pathway in macrophages might contribute to the elevated ceramide. Inhibition of the ceramide *de novo* synthesis pathway by myriocin markedly alleviated BAPN-induced aortic inflammation and dissection in mice. *In vitro* studies demonstrated that exogenous C18-ceramide promoted macrophage inflammation and matrix metalloprotein (MMP) expression through the NLRP3-caspase 1 pathway. In contrast, inhibition of endogenous ceramide synthesis by myriocin attenuated lipopolysaccharide (LPS)-induced macrophage inflammation.

**Conclusions:**

Our findings demonstrated that ceramide metabolism disturbance might play a vital role in TAD development by aggravating aortic inflammation through the NLRP3 pathway, possibly providing a new target for pharmacological therapy and a potential biomarker of TAD.

## Introduction

Thoracic aortic dissection (TAD) is usually an acute onset and life-threatening disease, characterized by any intimal tear in the thoracic aorta that allows blood flow to enter the aortic media. If untreated, its mortality is up to 33% within the first 24 h and rises to 50% by the first 48 h. An epidemiological survey of aortic dissection in China shows that the incidence rate in urban Chinese adults is 2.78 per 100,000 person-years (1). Current pharmacological therapy can only delay the expansion of aortic aneurysms rather than prevent aortic dissection or aneurysm rupture. To date, prophylactic surgical repair is so far the only effective strategy to avoid the occurrence of malignant events. Most of the time, preventive surgical intervention is recommended when the aorta diameter reaches 5.0–5.5 cm. However, studies have shown that up to 60% of acute type A aortic dissections occur when the diameter is <5.5 cm (2). Therefore, there remains an urgent need to explore new pathological mechanisms in the pathogenesis of TAD and biomarkers for timely surgical intervention.

In recent years, metabolomics has emerged as a useful tool for deciphering new disease pathogenesis and identifying novel biomarkers, as it situates downstream of physiological and pathological changes and therefore reflects cellular processes more directly and unbiasedly than genomic, transcriptomic, and proteomic variations. This approach has provided valuable insight into novel pathologic pathways and potential biomarkers in several cardiovascular diseases (3), such as cardiac hypertrophy, heart failure, coronary artery disease, and cardiovascular risk prediction. To the best of our knowledge, only a few studies (4–7) have focused on thoracic aortic diseases, and the findings have suggested that several bioactive lipids, such as glycerophospholipids, sphingolipids and lysophosphatidylcholines (7), as well as some other metabolites, such as fumarate (8) and succinate (9), are significantly altered in aortic disease.

Bioactive lipids can play a variety of roles in regulating cellular processes and functions, of which sphingolipids have emerged as molecules of special interest. In recent years, sphingolipids, especially their core metabolite ceramides, have attracted much attention for their association with cardiovascular diseases (10). Studies have shown that specific ceramides are positively correlated with the incidence of cardiovascular disease (11), secondary cardiovascular events (12), and mortality (13) and may become a potential biomarker for predicting the risk of atrial fibrillation (14) and poor prognosis of coronary artery disease (15). Although the exact mechanisms underlying these observations remain largely unknown, several studies have implicated that ceramdies might promote cardiovascular pathological progress through multiple pathways, such as exacerbating the inflammatory response (16–18), promoting cell apoptosis (17), and producing superoxide anions (16), as well as some other pathways (10).

In this study, we performed untargeted metabolomics analysis to delineate the metabolic landscape of patients with thoracic aortic aneurysm and dissection and explored potential biomarkers and novel mechanisms underlying TAD. The results indicated that the specific C18-ceramide was significantly increased in aortic dissection, but not in aortic aneurysm. Subsequently, we further investigated the source and pathological significance of excessive ceramides.

## Materials and Methods

### Human Subjects and Specimens

Syndromic and non-syndromic thoracic aneurysm and Stanford type A aortic dissection patients were enrolled from Fuwai Hospital between August 2017 and December 2020. Aneurysms and dissections were evaluated and diagnosed by echocardiography or computed tomography (CT). Thoracic aortic aneurysm (TAA) was defined as a localized dilation of the thoracic aorta that was more than 50% of predicted, and Stanford type A aortic dissection referred to dissections involving any part of the aorta proximal to the origin of the left subclavian artery. Patients with aortic trauma, pseudoaneurysm, or arteritis were excluded. Healthy control subjects were recruited from physical examination individuals at Fuwai Hospital, who had neither significant systemic disease nor aortic dilation evaluated by echocardiography. Two datasets were established to identify the plasma metabolite features. In the discovery cohort, 140 patients (70 TAA, 70 TAD) and age- and sex- matched healthy individuals were enrolled. The validation cohort included 269 aortic disease patients (183 TAAs, 86 TADs) with aortic diameters ≤ 5.5 cm. Blood samples were collected during outpatient service or after anesthesia induction and before heparin during surgery. 3–4 ml venous blood was collected in ethylene diamine tetraacetic acid (EDTA)-anticoagulated tubes, and plasma was separated by centrifuging at 3,000 rpm for 10 min at room temperature within 1 h and stored at −80°C until analysis.

This study conformed to the Declaration of Helsinki principles and was approved by the ethics committee of the institutional review board at Fuwai Hospital (Approval No.:2017-877). All of the patients and healthy control subjects included in this study assigned a consent form.

### Untargeted Metabolomics Analysis

Plasma metabolomics was performed by Metabolon (Durham, NC, USA) using a multiplatform system encompassing tandem ultrahigh-performance liquid chromatography-mass spectrometry as well as tandem gas chromatography-mass spectrometry systems with different column ionization parameters.

Plasma samples were prepared using the automated MicroLab STAR system (Hamilton Company). Several recovery standards were added before the first step in the extraction process for quality control purposes. Plasma proteins were precipitated with methanol under vigorous shaking for 2 min (Glen Mills GenoGrinder 2000) followed by centrifugation. The resulting extract was divided into five fractions: two for analysis by two separate reverse phase/ultra-performance liquid chromatography (UPLC)-tandem mass spectrometry (MS/MS) methods with positive ion mode electrospray ionization (ESI), one for analysis by reverse phase/UPLC-MS/MS with negative ion mode ESI, one for analysis by hydrophilic interaction liquid chromatography (HILIC)/UPLC-MS/MS with negative ion mode ESI and one sample reserved for backup. Samples were placed briefly on a TurboVap (Zymark) to remove the organic solvent. The sample extracts were stored overnight under nitrogen before preparation for analysis.

Several types of controls were analyzed in concert with the experimental samples: a pool of well-characterized human plasma served as a technical replicate throughout the dataset; extracted water samples served as process blanks; and a cocktail of quality control standards that were carefully chosen not to interfere with the measurement of endogenous compounds were spiked into every analyzed sample, allowed instrument performance monitoring and aided chromatographic alignment. Instrument variability was determined by calculating the median relative s.d. for the standards that were added to each sample before injection into the mass spectrometers. Overall process variability as determined by calculating the median relative s.d. for all endogenous metabolites (that is, non-instrument standards) present in 100% of the pooled matrix samples was 10%. Experimental samples were randomized across the platform run with quality control samples spaced evenly among the injections.

All methods utilized an ACQUITY ultra-performance liquid chromatography (UPLC; Waters, Milford, MA, USA), a Q-Exactive high resolution/accurate mass spectrometer interfaced with a heated electrospray ionization (HESI-II) source (Thermo Scientific, Waltham, MA, USA) and Orbitrap mass analyzer operated at 35,000 mass resolution. The sample extract was dried and reconstituted in solvents compatible with each of the four methods. Each reconstitution solvent contained a series of standards at fixed concentrations to ensure injection and chromatographic consistency. One aliquot was analyzed using acidic positive ion conditions, chromatographically optimized for more hydrophilic compounds. The extract was gradient eluted from a C18 column (Waters UPLC BEH C18−2.1 × 100 mm, 1.7 μm) using water and methanol, containing 0.05% perfluoropentanoic acid (PFPA) and 0.1% formic acid. Another aliquot was also analyzed using acidic positive ion conditions, which was chromatographically optimized for more hydrophobic compounds. The extract was gradient eluted from the C18 column using methanol, acetonitrile, water, 0.05% PFPA and 0.01% formic acid and was operated at an overall higher organic content. Another aliquot was analyzed using basic negative ion optimized conditions using a separate dedicated C18 column. The basic extracts were gradient eluted from the column using methanol and water with 6.5 mM Ammonium Bicarbonate at pH 8. The fourth aliquot was analyzed *via* negative ionization following elution from a HILIC column (Waters UPLC BEH Amide 2.1 × 150 mm, 1.7 μm) using a gradient consisting of water and acetonitrile with 10 mM ammonium formate, pH 10.8. The MS analysis alternated between MS and data-dependent MSn scans using dynamic exclusion. The scan range covered 70–1,000 m/z.

Raw data were extracted, peak-identified and QC processed by Metabolon (Metabolon, Inc., Morrisville, NC, USA). Compounds were identified by comparison to library entries of purified standards or recurrent unknown entities. Biochemical identifications are based on three criteria: retention index within a narrow RI window of the proposed identification, accurate mass match to the library ±10 ppm, and the MS/MS forward and reverse scores between the experimental data and authentic standards. The MS/MS scores are based on a comparison of the ions present in the experimental spectrum to the ions present in the library spectrum. Peaks were quantified by Metabolon, Inc. using the area-under-the-curve with a data normalization step was performed to correct variation resulting from instrument inter-day tuning differences.

Statistical analysis was performed by Metabolon as well as using MetaboAnalyst 5.0 (https://www.metaboanalyst.ca, accessed date on 6 June 2021) for pathway and enrichment analyses. Following log transformation and imputation of missing values, if any, with the minimum observed value for each compound, Welch's two-sample *t*-test was used to identify biochemicals that differed significantly between experimental groups, while outliers were identified using the ROUT method. Biochemicals that achieved statistical significance (*p* ≤ 0.05) and demonstrated a low estimate of false discovery rate (*q* > 0.10) were included in this analysis.

Statistical analysis was performed by Metabolon as well as using online MetaboAnalyst 5.0 (https://www.metaboanalyst.ca) for pathway and enrichment analyses. Following log transformation and imputation of missing values, if any, with the minimum observed value for each compound, Welch's two-sample *t*-test was used to identify biochemicals that differed significantly between experimental groups, while outliers were identified using the ROUT method. Biochemicals with fold change (FC) > 1.3 or FC <1/1.3 that achieved statistical significance (*p* ≤ 0.05) and demonstrated a low estimate of false discovery rate (*q* > 0.10), were included in this analysis.

### Quantitative Measurement of Plasma C18-Ceramide

The SHIMADZU Prominence UPLC system (Kyoto, JAPAN) equipped with an Applied Biosystems 4500 Q-Trap mass spectrometer (Foster City, CA, USA) with electrospray ionization source was used. Chromatographic separation was achieved by using a Phenomenex Kinetex C8 column, 50 mm × 2.1 mm, 5 μm (phenomenex, USA). Mobile phase A was made of a 2-mM ammonium acetate aqueous solution with 0.1% formic acid by volume and mobile B 70:30 (v:v) acetonitrile: isopropanol with 0.1% formic acid by volume. The column temperature was set at 40°C, and the flow rate was 0.3 ml/min. The gradient elution program was set as follows, 0–1 min 65% B; 1–1.5 min 65–75% B; 1.5–3.5 min 75–95% B; 3.6–4.5 min 65% B.

The mass detection and quantification was performed in a positive electrospray ionization mode using multiple reactions monitoring (MRM) method. The mass spectrometer working parameters were optimized as follows: curtain gas at 35 psi, Gas 1 at 55 psi, Gas 2 at 55 psi and a turbo ion spray temperature of 550°C. The MRM transitions for C18-ceramide and the isotope internal standard C18-ceramide-d7 was 548.5–264.5 and 555.5–271.5. Data acquisition and procession was performed with Analyst 1.6.1 software version (AB SCIEX, Concord, Ontario, Canada).

To get the precise concentration, a standard curve was performed. An aliquot of 100 ul various concentration standards (10–500 ng/ml) were analyzed with the same procedure. The calibration curve was constructed by plotting the peak area ratios of each analyte/IS vs. its nominal concentrations in standards, using a linear regression. Standard curves were acceptable when coefficient of determination (R2) exceeded 0.990. Five replicates (*n* = 5) of QC samples were analyzed in the same batch assay to determine the intra-day precision and accuracy and in three different batch assays to determine the inter-day precision and accuracy of the method. The precision is expressed by relative standard deviation (RSD) between the replicate measurements. Accuracy is defined as relative error (RE) which is calculated using the formula RE% = [(measured value–theoretical value)/ theoretical value] × 100. The intra-day precision (RSD) ranged between 1.08 and 13.20%, and accuracy (RE) ranged between −11.83 and 5.78%. The inter-day precision (RSD) ranged between −10.89 and 2.11%, and accuracy (RE) ranged from 0.05 to 11.10%, respectively. All inter- and intra-day precision and accuracy were acceptable for working in biological media.

The quantitative detection limit (LOQ) of the method was investigated by detecting the linear lowest point of the standard curve. The lowest-concentration standard (10 ng/ml) was tested for 6 consecutive times, and coefficient of variation (CV) and accuracy (RE) were 4.00 and −1.17%, respectively, which was acceptable.

### Animal Model and Treatment

Wild-type (WT) mice on a C57B/L6 background were obtained from Vitalstar in Beijing. Three-week-old male mice were fed a normal diet and administered with freshly prepared β-aminopropionitrile (BAPN) solution (0.5 g/kg/day) dissolved in the drinking water for 4 weeks. Myriocin (0.5 mg/kg; APExBIO Inc.) or its solution was injected intraperitoneally from the beginning of BAPN administration every other day for 4 weeks. Then the mice were anesthetized to harvest the aortas. Anesthetization and euthanasia were performed by intraperitoneal injection of sodium pentobarbital (50 and 150 mg/kg, respectively).

All animal studies conformed to the NIH Guide for the Care and Use of Laboratory Animals and were approved by the Institutional Animal Care and Use Committee (IACUC) at Fuwai Hospital (Approval No.: FW-2019-0008).

### HE Stating and Elastin Van Geison Staining

HE staining and elastin Van Geison (VG) staining were conducted using kits (Zhongshan Golden Bridge Biotechnology, Beijing, China) according to routine protocols. Briefly, for HE staining, after deparaffinization and rehydration, aorta sections were stained with hematoxylin solution for 3–5 min followed by 5 dips in 1% acid ethanol (1% HCl in 70% ethanol) and then rinsed in distilled water. Then, the sections were stained with eosin solution for 3 min, dehydrated with graded alcohol and cleared in xylene. For elastin VG staining, potassium permanganate was oxidized for 5 min and bleached with oxalic acid for 5 min. Then, the sections were washed with 95% alcohol and stained with elastin dyeing at room temperature for 8–24 h. Differentiation was then performed with 95% alcohol or 1% hydrochloric acid, followed by washing with distilled water. Contrasting staining was performed with Van Gieson staining for 1 min, and rapid differentiation was performed with 95% alcohol for a few seconds. Then 100% alcohol dehydration was performed, followed by the use of transparent xylene and sealing with neutral gum.

### Immunohistochemistry (IHC)

Tissues were fixed in 4% paraformaldehyde (PFA) and embedded in paraffin. Six-μm sections were deparaffinized and rehydrated in an ethanol series, and antigen retrieval was performed in a steamer. Non-specific binding was blocked by 5% BSA in PBS. The sections were probed with primary antibody, washed and then probed with secondary antibody. Primary antibody: CD68 (Abcam, ab201340). IHC staining was developed using DAB (Vector Laboratories) followed by hematoxylin counterstaining. After mounting, the slips were visualized by microscopy.

### Cell Culture and Treatment

RAW 264.7 cells were cultured in Dulbecco's modified Eagle's medium (DMEM; Gibco, 10569044) supplemented with 10% fetal bovine serum (FBS), 100 U/mL penicillin and 100 μg/mL streptomycin and incubated at 37°C under 5% CO_2_. For ceramide treatment, cells were starved for 24 h and then primed by 100 ng/ml LPS for 16 h. After the pretreatment, cells were incubated in Cer18 (GlpBio Inc.) at a concentration of 10 μmol/L for 24 h. For LPS and myriocin treatment, cells were starved for 24 h and then incubated in 1 μg/ml LPS (Sigma) with or without 10 μmol/L myriocin.

### RNA Isolation and Quantitative Real-Time PCR

Total RNA from aortic tissues or RAW264.7 cells was extracted with TRIzol reagent (Invitrogen, 15596018). Real-time PCR samples were prepared by mixing cDNAs, power-SYBR Mix (ABI, A25742) and specific primer sets (Supplementary Table 1). The initial denaturation step of PCR amplification was 95°C for 10 min, followed by 40 cycles of 95°C for 15 s and 55°C for 1 min, then 95°C for 15 s and 60°C for 1 min, and last at 95°C for 1 s. Gene expressions were normalized against *Gapdh*.

### Western Blot

Cells were lysed with RIPA (Beyotime P0013B) lysis buffer. Equal amounts of protein were separated by SDS–PAGE, transferred to nitrocellulose membranes, blocked in 5% skimmed milk, and incubated with the primary antibodies against NLRP3 (1:1000, CST 15101S), Caspase1 (1:150,), IL-1β (1:500, CST), MMP9 (1:500, Abcam), and β-actin (1:50000, Proteintech) overnight at 4°C, followed by detection with Horseradish enzyme labeled goat anti-rabbit IgG (H+L; ZSGB-BIO, ZB-2301). Visualization was performed with Chemiluminescence imaging system (Bio Rad, ChemiDocXRS+).

### Statistical Analysis

Data were analyzed using GraphPad Prism version 5.01 (GraphPad Software, San Diego, CA) and SPSS version 22.0 (IBM SPSS Statistics, Armonk, NY). The continuous variables were described as the means and standard deviations (SDs) with a normal distribution or as medians and interquartile ranges (IQRs) with an abnormal distribution, and the categorical variables were described as proportions. Statistical analysis was performed by unpaired or paired *t*-test for two groups of data and by one-way ANOVA for multiple comparisons. Statistical significance among multiple groups was determined by *post-hoc* analysis (Tukey honestly significant difference test). Proportions were compared using the Chi-square test. Values of *p* < 0.05 were considered statistically significant.

## Results

### Global Metabolic Profiles Revealed a Significant Increase in C18-Ceramide in TAD Patient Plasma and Quantitative Analysis Verified It

**Figure 1:**
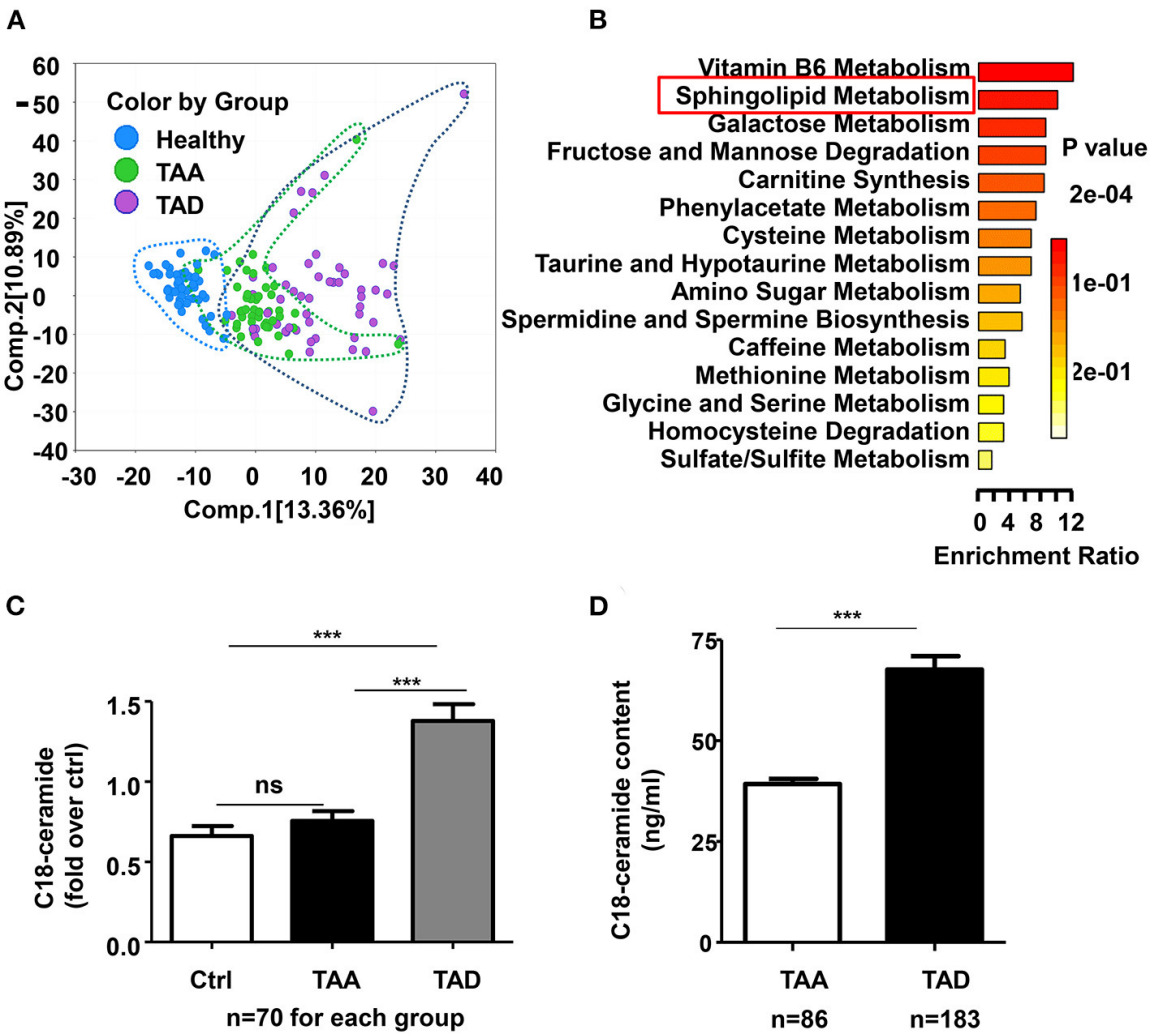
The global metabolic profile revealed a significant change in sphingolipid metabolism, especially C18-ceramide in TAD patients. **(A)** Principal component analysis (PCA) demonstrated distinguished metabolic signatures among TAA, TAD and healthy controls. **(B)** Metabolite set enrichment analysis showed the top 15 significantly altered enrichment pathways between TAAs and TADs. **(C)** Untargeted metabolomics result demonstrated that C18-ceramide was significantly elevated in TAD patients but not significantly changed in TAA patients (ctrl, *n* = 70; TAA, *n* = 70; TAD, *n* = 70), One-way ANOVA, ***p < 0.001. **(D)** Quantitative analysis confirmed the increase in C18-ceramide in early-onset TAD patients (TAA, *n* = 183; TAD, *n* = 86), unpaired *t*-test, ***p < 0.001.

To explore novel mechanisms and potential biomarkers of thoracic aortic aneurysm/dissection, 70 thoracic aortic aneurysm (TAA) patients and 70 type A aortic dissection (TAD) patients, as well as 70 age- and sex- matched controls, were subjected to untargeted metabolomics by using ultrahigh performance liquid chromatography-tandem mass spectrometry (UPLC–MS/MS) method (Metabolon, USA). The demographic and clinical information of all patients and healthy controls was listed in (Supplementary Table 2). A total of 866 named biochemicals in plasma were determined. Principal component analysis (PCA) revealed that both TAA and TAD separated from controls, but TAD showed more distant separation (Figure 1A), suggesting that TAA and TAD display related metabolic signatures, with TAD exhibiting the more extreme metabolic phenotype.

A total of 373 significantly differentiated metabolites were observed between TAA and TAD (Supplementary Table 3), of which 145 were elevated while 228 were decreased. Pathway analysis of differentiated metabolites between TAA and TAD was presented in Supplementary Figure 1. Metabolite set enrichment analysis demonstrated that sphingolipid metabolism was one of the most distinguished enriched pathways (Figure 1B). Notably, its core metabolite, N-stearoyl-sphingosine (d18:1/18:0; C18-ceramide), was ~2-fold higher in TAD patients than in healthy controls, but not significantly changed in TAA patients (Figure 1C), suggesting that it might be a distinguished biomarker between TAA and TAD. After excluding the 10 TAD patients who had already a history of aortic surgeries before the dissection onset, the remaining 60 TAD patients were divided into three groups based on the tirtile valule of C18-ceramide relative amount (low, moderate, high) and we observed that the rate of early-onset dissection in moderate- and high-level ceramide group was higher than in low-level group (Table 1) (90, 85 vs. 45%, *n* = 20, *p* = 0.002).

**Table 1:** The proportion of early-onset TAD in each group of TAD patients with different amount of C18-ceramide level.

	**C18-ceramide group**			
	**Low**	**Moderate**	**High**	***p*-value**
Numbers	20	20	20	
Age (year-old)	52 (39–62.75)	54.5 (47.25–64.75)	49 (43–57.25)	0.2926
Male (%)	11 (55%)	12 (60%)	16 (80%)	0.2147
Hypertension (%)	14 (70%)	16 (80%)	16 (80%)	0.689
Hyperlipidemia (%)	12 (60%)	12 (60%)	15 (75%)	0.5171
Diabetes (%)	0 (0%)	1 (5%)	3 (15%)	0.1534
Early onset aortic dissection	9 (45%)	17 (85%^**^)	18 (90%^**^)	0.0020

** *p < 0.01 when compared to the low-level C18-ceramide group*.

To confirm the association between C18-ceramide content and ealry-onset aortic dissection, we subsequently enrolled 269 TAAD patients (183 TAA and 86 TAD) with aortic diameters ≤ 5.5 cm and quantitatively detected their plasma C18-ceramide content. The demographic and clinical information of all patients was listed in Supplementary Table 4. The results showed that C18-ceramide concentrations were elevated in ealry-onset TAD patients (66.3 ± 30.9 ng/ml, *n* = 86) compared with those in TAA patients (39.3 ± 16.3 ng/ml, *n* = 183) (Figure 1D).

### Multiomics Data Suggested That Macrophage-Derived Ceramide *de novo* Synthesis Contributed to the Increased Ceramide Content

**Figure 2:**
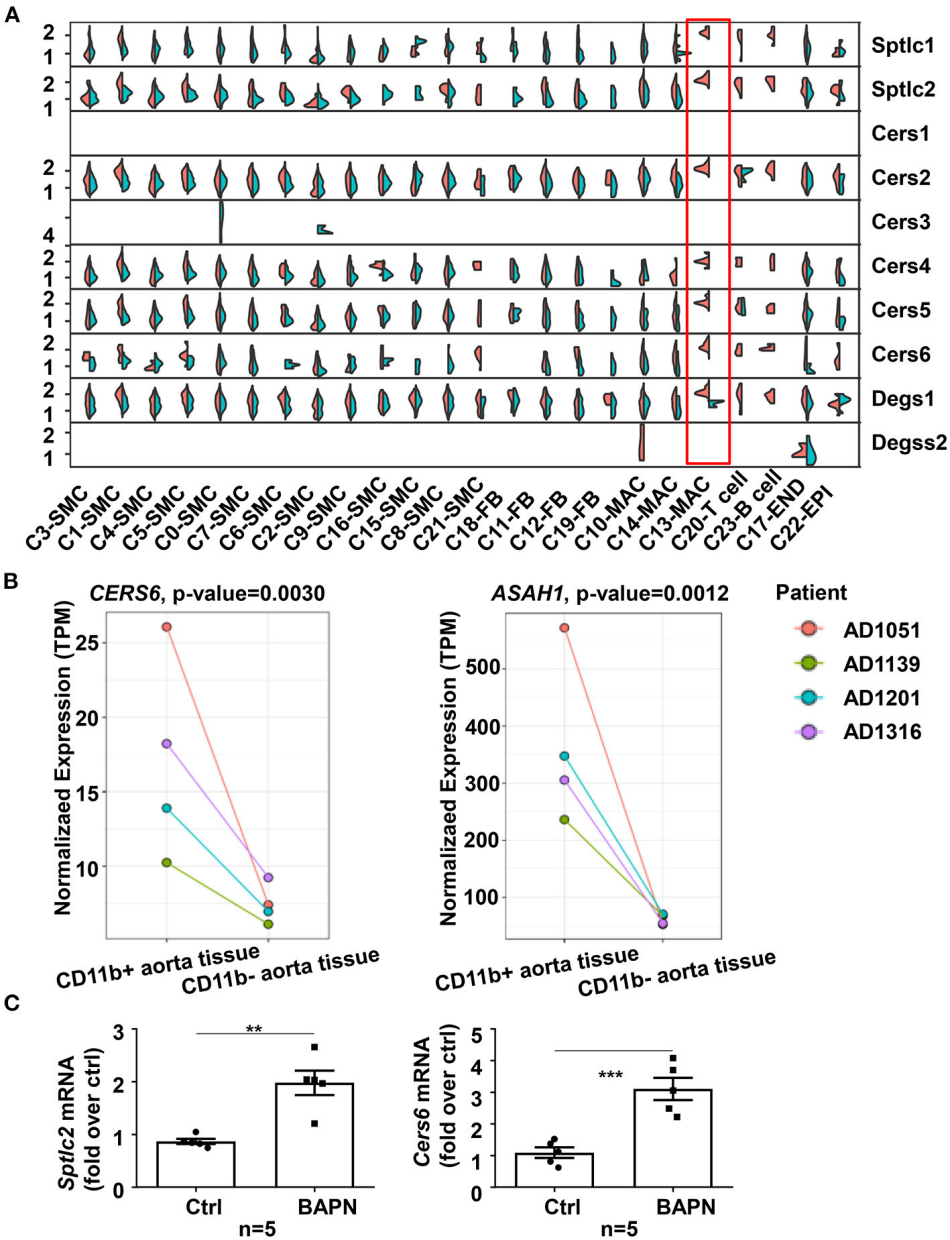
Enhanced ceramide *de novo* synthesis in macrophages contributed to the increased ceramide content in plasma. **(A)** Split violin plot showed the expression of genes involved in ceramide synthesis pathways in mouse aortic cell subpopulations of the BAPN and CTRL groups in the 21-day mouse model. **(B)** Expression of ceramide synthesis and hydrolysis genes, *CERS6* and *ASAH1*, was higher in CD11b^+^ aortic cells from TAAD patients than in CD11b^−^ cells (*n* = 4). The statistical threshold of the differential expression test was set to be a *q*-value <0.05. **(C)** Expression of ceramide *de novo* synthesis genes, *Sptlc2* and *Cers6*, was elevated in aortas from BAPN-induced TAD mice (*n* = 5), unpaired *t*-test, **p < 0.01, ***p < 0.001.

To investigate the source of elevated ceramide in plasma in TAD, we tried to find clues from our two existing RNA-sequencing (RNA-seq) datasets (19). In our previous study, we had performed time-series single-cell RNA-seq of thoracic aortic cells from β-aminopropionitrile (BAPN)-induced TAAD mouse models. Genes in the ceramide *de novo* synthesis pathway, such as *Sptlc, Cers*, and *Degs*, were significantly upregulated in the proinflammatory macrophage subpopulation c13 (*Il*1*rn*^+^) in BAPN-induced mice for 21 days (Figure 2A). Genes in the sphingomyelin hydrolysis and salvage pathways were not significantly altered (data not shown). In addition, our other aortic bulk RNA-Seq dataset from humans demonstrated that higher expression of *CERS6* (ceramide synthesis gene) and *ASAH1* (ceramide hydrolysis gene) was observed in CD11b^+^ macrophages than CD11b^−^ cells in aortic tissues from TAAD patients (Figure 2B). Combining these two RNA-seq results, we speculated that elevated ceramide content was due to an enhanced ceramide *de novo* synthesis pathway in aortic macrophages. Furthermore, we quantified the expression of all major genes involved in ceramide synthesis in aortic tissues from TAD mice and found that only *de novo* pathway genes, *Sptlc2* and *Cers6*, were significantly upregulated under BAPN-induced TAD conditions (Figure 2C).

### Inhibition of Ceramide *de novo* Synthesis With Myriocin Prevented BAPN-Induced Thoracic Aortic Dissections and Aortic Inflammation in Mice

**Figure 3:**
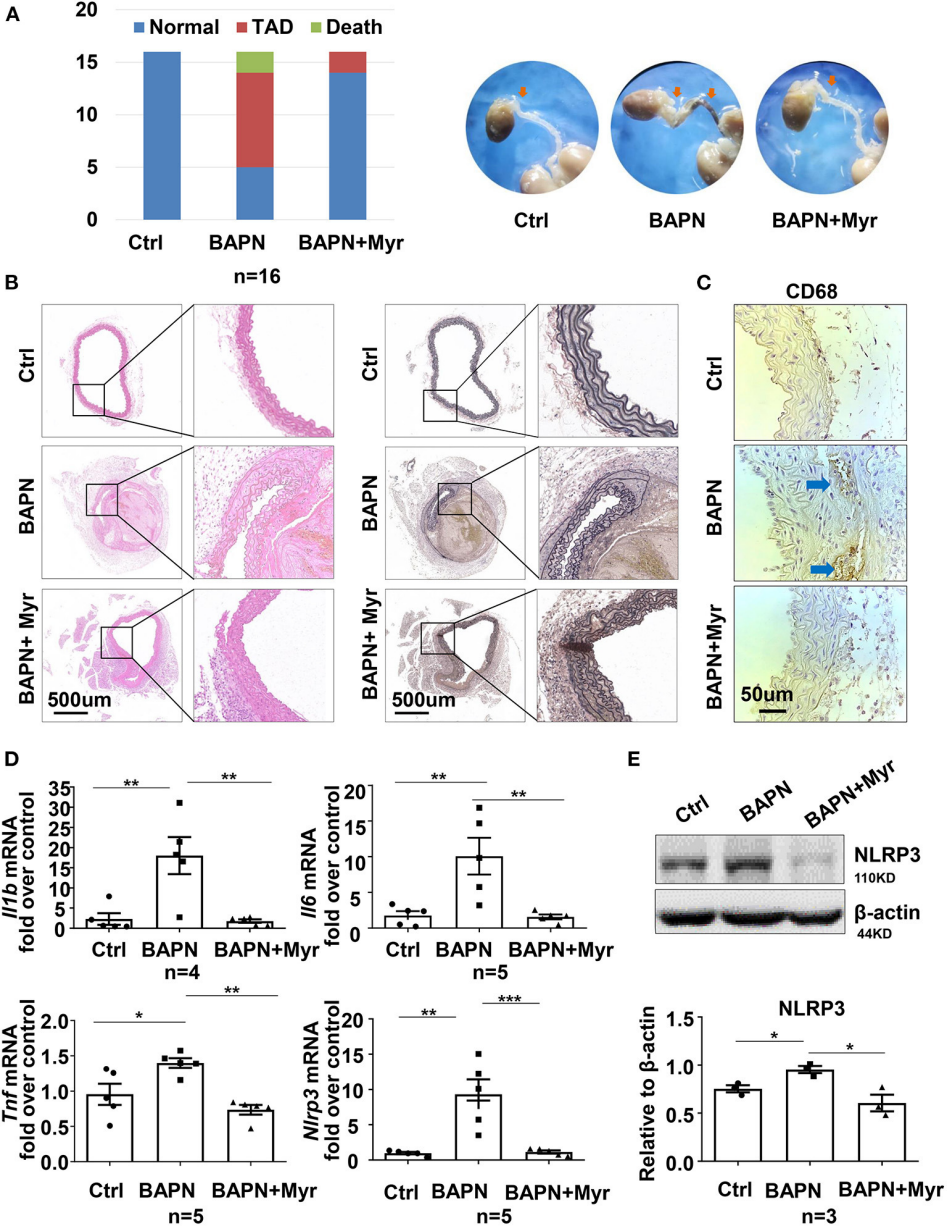
Inhibition of ceramide *de novo* synthesis with myriocin alleviated BAPN-induced aortic dissection and inflammation. Three-week-old mice were administered with BAPN (0.5 g/kg/day) for 4 weeks, with or without intraperitoneally injected myriocin (0.5 mg/kg). **(A)** Incidence of TAD events and related death for each group and representative aorta images (*n* = 16). **(B)** Representative HE and VG staining images for each group (*n* = 3). **(C)** Representative immunohistochemical staining for macrophage marker CD68 (*n* = 3). **(D)** Quantification of the mRNA expression of *Il1b, Il6*, Tnf, and *Nlrp3* in the aorta by RT-qPCR (*n* = 5), One-way ANOVA, *p < 0.05, **p < 0.01, ***P < 0.001. **(E)** Representative western blot of NLRP3 in the aorta (*n* = 3), One-way ANOVA, *p < 0.05. Myr, myriocin.

To determine the role of ceramide in TAD, we examined the effect of the ceramide *de novo* synthesis inhibitor myriocin on a BAPN-induced TAD mouse model. Forty-eight mice were randomly divided into three groups: WT, BAPN, and BAPN+myriocin. The results showed that BAPN-induced aortic dissection and related death occurred in 68.75% (11/16) of mice, while the rate in the BAPN+myriocin group was 12.5% (2/16), suggesting that myriocin effectively prevented BAPN-induced TAD (Figure 3A). HE and elastin VG staining staining demonstrated that myriocin mitigated BAPN-induced aortic damage and elastic fiber degradation (Figure 3B). Histochemical staining of CD68^+^ in the aorta revealed that BAPN promoted the accumulation of macrophages and that myriocin suppressed this phenomenon (Figure 3C). Moreover, myriocin alleviated BAPN-induced aortic inflammatory cytokine (IL-1β, TNF-α, and IL-6) expression and NLRP3 inflammasome-related gene expression and protein (Figures 3D,E). Altogether, the present results suggested that inhibition of ceramide *de novo* synthesis could alleviate BAPN-induced aortic inflammation and dissection.

### Ceramide Accelerated Inflammation Through the NLRP3-Caspase1 Pathway in Macrophages

**Figure 4:**
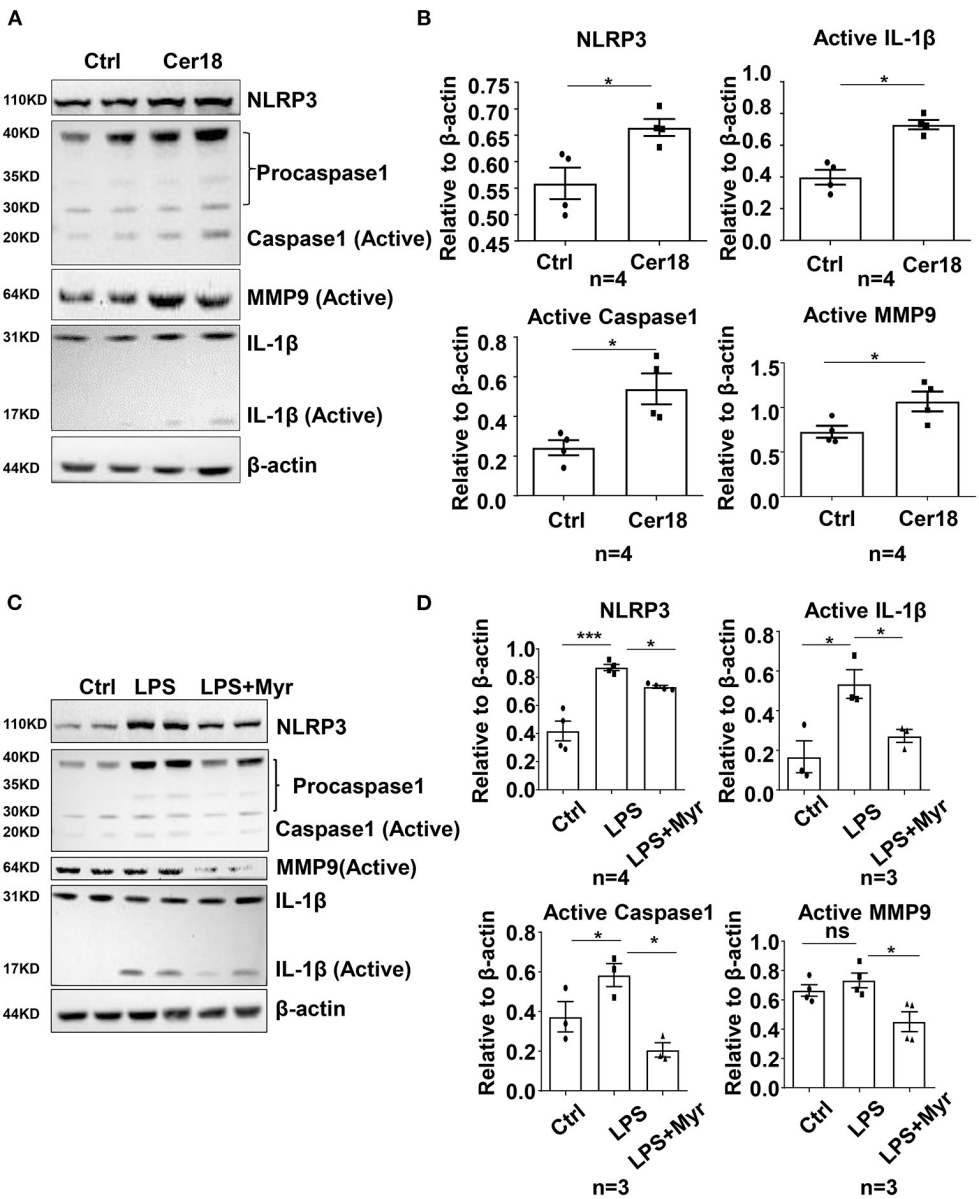
Ceramide was involved in the activation of IL-1β and MMP9 by the NLRP3-caspase1 cascade in mouse macrophage RAW264.7. **(A,B)** Representative western blots and their quantification showed that C18-ceramide induced the activation of IL-1β and MMP9 by the NLRP3-caspase1 cascade, *n* = 4, unpaired *t*-test, *p < 0.05, **p < 0.01, ***p < 0.001. **(C,D)** Representative western blots and their quantification showed that myriocin prevented LPS-induced the activation of IL-1β and MMP9 by the NLRP3-caspase1 cascade, *n* ≥ 3, One-way ANOVA, *p < 0.05, **p < 0.01, ***p < 0.001. Myr, myriocin; Cer18, C18-ceramide.

To further investigate the underlying mechanism of ceramide in inflammation, we performed *in vitro* studies in the mouse macrophage cell line RAW264.7. Treatment of RAW 264.7 cells with exogenous C18-ceramide at 10 μmol/L for 24 h upregulated the expression of NLRP3, Caspase 1, Il1b, and Mmp9 and coincidingly promoted IL-1β and MMP9 cleavage (Figures 4A,B). Conversely, myriocin impeded LPS-induced NLRP3–caspase 1 cascade activation and subsequent IL-1β and MMP9 cleavage (Figures 4C,D).

## Discussion

TAD is often a life-threatening condition with high morbidity and mortality, and is characterized by smooth muscle cell loss, extracellular matrix degradation and increased vascular inflammation. Metabolomics has developed rapidly and offers an opportunity to explore new advances and potential biomarkers underlying TAD. Several lipid species alterations, such as increased phosphatidylcholine (4, 6) and decreased lysophosphatidylcholines and sphingolipids (5), were observed in aortic dissection patients and were regarded as potential biomarkers. However, these studies had some limitations. First, they had relatively small datasets and the results were not verified in larger cohorts. Second, they were observational studies, and the causal relationship between altered metabolites and the disease was not investigated. Therefore, the relationship needs to be further confirmed. In the present study, we aimed to explore the differentiated metabolites in TAA and TAD patients using an untargeted metabolomics approach. To our knowledge, this was the largest untargeted metabolomic study on thoracic aortic disease so far. It allowed for investigating the distinguished metabolites between TAA and TAD, so as to explore the specific pathophysiological process in TAD and potential biomakers.

From metabolite set enrichment analysis of differentiated metabolites between TAA and TAD, we observed vitamin B6 metabolism was the most enriched pathway. Its metabolite pyridoxate was significantly decreased in TAA, but not in TAD (Supplementary Table 3). Vitamin B was reported to be associated with abdominal aortic aneurysm (20) and to mitigate thoracic aortic dilation in Marfan syndrome mice (21). Whether it was involved in aortic dissection remained unclear. Beyond that, we were more interested in another enriched pathway, sphingolipids, which were recently focused as a variety of cardiovascular diseases drivers and biomarkers. Consistent with previous studies, most sphingolipids including various sphingomyelins were significantly decreased in TAD (Supplementary Figure 2) (5, 7). However, we noticed significantly increased C18-ceramide content in the plasma of TAD patients, but not in TAA, from which we hypothesized that ceramide might play a role in TAD incidence.

Ceramides are the central core of sphingolipid metabolism. They can be synthesized by different pathways: (1) the *de novo* synthesis pathway; (2) the complex sphingolipid (such as sphingomyelin) hydrolysis pathway; and (3) the salvage pathway (22). The former two contribute mostly to ceramide production. Once generated, ceramides can participate in numerous physiological and pathological processes, such as cellular proliferation and migration, inflammatory responses, apoptosis and senescence. Our existing single-cell RNA-Seq data from a murine aortic dissection model revealed specifically high expression of *Sptlc, Cers*, and *Degs* in a subpopulation of macrophages, suggesting that elevated ceramide might contribute to the enhanced *de novo* synthesis pathway in macrophages. Therefore, we focused on the inflammatory impact of ceramide in aortic dissection.

Aortic dissection is closely related to inflammatory processes. A variety of inflammatory cells can invade the vascular wall and cause arterial inflammation, among which macrophages are the core cells (23). Activated macrophages secrete matrix metalloproteinases (MMPs), interleukins and vascular endothelial cell growth factors to further magnify the inflammatory response, matrix destruction and smooth muscle cell dysfunction, resulting in aortic injury and ultimately aortic dissection. After dissection, a large number of inflammatory cells enter the site of dissection, aggravating the local inflammatory response and participating in later aortic remodeling. Lian et al. (8) and Cui et al. (9) Both revealed that macrophage metabolic reprogramming was involved and played a vital role in aortic dissection. Some metabolites in the tricarboxylic acid (TCA) cycle, such as fumarate (8) and succinate (9), were substantially elevated in aortic dissection patients and aortic dissection mouse models induced by BAPN or AngII stimulation. Reprogrammed macrophages exaggerated vascular inflammation through the HIF1α-ADAM17 pathway and excessive ROS production, ultimately leading to aortic dissection.

Ceramides can promote inflammation through a variety of pathways (24). They can induce the expression or activation of NF-κB, a ubiquitous transcription factor involved in inflammatory and immune responses, and then upregulate many proinflammatory genes, including the cytokine genes IL-1β, IL-6, and IL-8, and chemokine genes, such as monocyte chemoattractant protein-1 (MCP-1). Deficiency of serine palmitoyltransferase subunit 2, a key enzyme involved in the ceramide *de novo* synthesis pathway, could reduce murine atherosclerosis partly by attenuating TLR4 recruitment and downstream NF-κB-mediated inflammation (25). In addition, the NOD-like receptor pyrin domain containing 3 (NLRP3) inflammasome is another important pathway that is involved in ceramide-mediated inflammation. Ceramides activate the Nlrp3 inflammasome and IL-1β secretion through caspase-1 activation in a Nlrp3-dependent manner in various tissues, including adipose (26), thymus (27) and brain microglia (28), suggesting that the Nlrp3 inflammasome could sense intracellular elevated ceramide.

Recent studies have suggested that the NLRP3 inflammasome plays an important role in TAD development (29–31). The Nlrp3-caspase 1 complex is activated in the condition of aortic dissection and participates in the process of aortic damage by degrading smooth muscle cell contractile protein (29), promoting macrophage inflammation and *MMP9* expression and intensifying extracellular matrix degradation. The NLRP3 inhibitor MCC950 effectively prevented aortic aneurysm and dissection induced by AngII in mice (30). However, the relationship between ceramide and inflammasome activation in TAD development remains unknown. Therefore, the present study sought to determine whether ceramide promotes TAD development by inducing NLRP3 inflammasome formation.

Our *in vivo* study demonstrated that inhibition of ceramide *de novo* synthesis by myriocin significantly alleviated BAPN-induced aortic dissection, and NLRP3 mRNA and protein levels were both decreased in BAPN-treated mouse aortae. Consistently, *in vitro* studies in the murine macrophage cell line RAW264.7 indicated that myriocin impeded the LPS-induced NLRP3-caspase 1 pathway, while exogenous C18-ceramide promoted this pathway, suggesting that the NLRP3-caspase 1 pathway might be involved in ceramide-induced inflammation and aortic injury.

Nevertheless, our present study had some limitations. First, it was still a cross-sectional study, and further investigations in prospective and longitudinal cohorts were needed to confirm the association between ceramide and early-onset aortic dissection risk. Second, ceramides in aortic tissues in human and mice were neither determined in this study. Further, alleviating BAPN-induced TAD through inhibition of ceramide *de novo* synthesis pathway might be better proved using *Sptlc2* deficient mouse models.

In summary, our current study first revealed that ceramide metabolism disturbance might play a vital role in TAD development by aggravating aortic inflammation through the NLRP3 pathway, possibly providing a new target for pharmacological therapy and a potential biomarker of TAD.

## Data Availability Statement

The original contributions presented in the study are included in the article/Supplementary Material, further inquiries can be directed to the corresponding author/s. The scRNA-seq data presented in the study are deposited in the Genome Sequence Archive (http://bigd.big.ac.cn/gsa/), accession number CRA003013.

## Ethics Statement

The studies involving human participants conformed to the Declaration of Helsinki principles and were reviewed and approved by Ethics Committee of the Institutional Review Board at Fuwai Hospital (Approval No.: 2017-877). The patients/participants provided their written informed consent to participate in this study. The animal study was reviewed and approved by Institutional Animal Care and Use Committee (IACUC) at Fuwai Hospital (Approval No.: FW-2019-0008). Written informed consent was obtained from the individual(s) for the publication of any potentially identifiable images or data included in this article.

## Author Contributions

HY designed the project, carried out experiments, and drafted the manuscript together with FY. ML and CS were in charge of patient enrollment and clinical evaluation. QC and YZ participated in clinical data collection and the follow-up study. XL contributed to bioinformatics analysis of existing RNA-seq data. GZ, WC, and TL participated in animal experiments and cell experiments, respectively. ZZ was in charge of the project design and revised the manuscript. All authors had read and approved the final manuscript.

## Funding

This work was supported by the grant of CAMS Initiative for Innovative Medicine, China (No. 2016-I2M-1-016) and the grant of Initiative Research Program from State Key Laboratory of Cardiovascular Disease (SKL2021008).

## Conflict of Interest

The authors declare that the research was conducted in the absence of any commercial or financial relationships that could be construed as a potential conflict of interest.

## Publisher's Note

All claims expressed in this article are solely those of the authors and do not necessarily represent those of their affiliated organizations, or those of the publisher, the editors and the reviewers. Any product that may be evaluated in this article, or claim that may be made by its manufacturer, is not guaranteed or endorsed by the publisher.
