# Supplementary material for: Metabolomic Profile Reveals That Ceramide Metabolic Disturbance Plays an Important Role in Thoracic Aortic Dissection
Source: Front Cardiovasc Med. 2022 Feb 8;9:826861. doi: 10.3389/fcvm.2022.826861 (PMC8861291; doi:10.3389/fcvm.2022.826861)
Supplement: Supplementary file 1 [file Data_Sheet_1.docx]

Supplementary Material

# Supplementary Figures and Tables

## Supplementary Figures

Fig. S1. Pathway analysis of differentiated metabolites between TAA and TAD


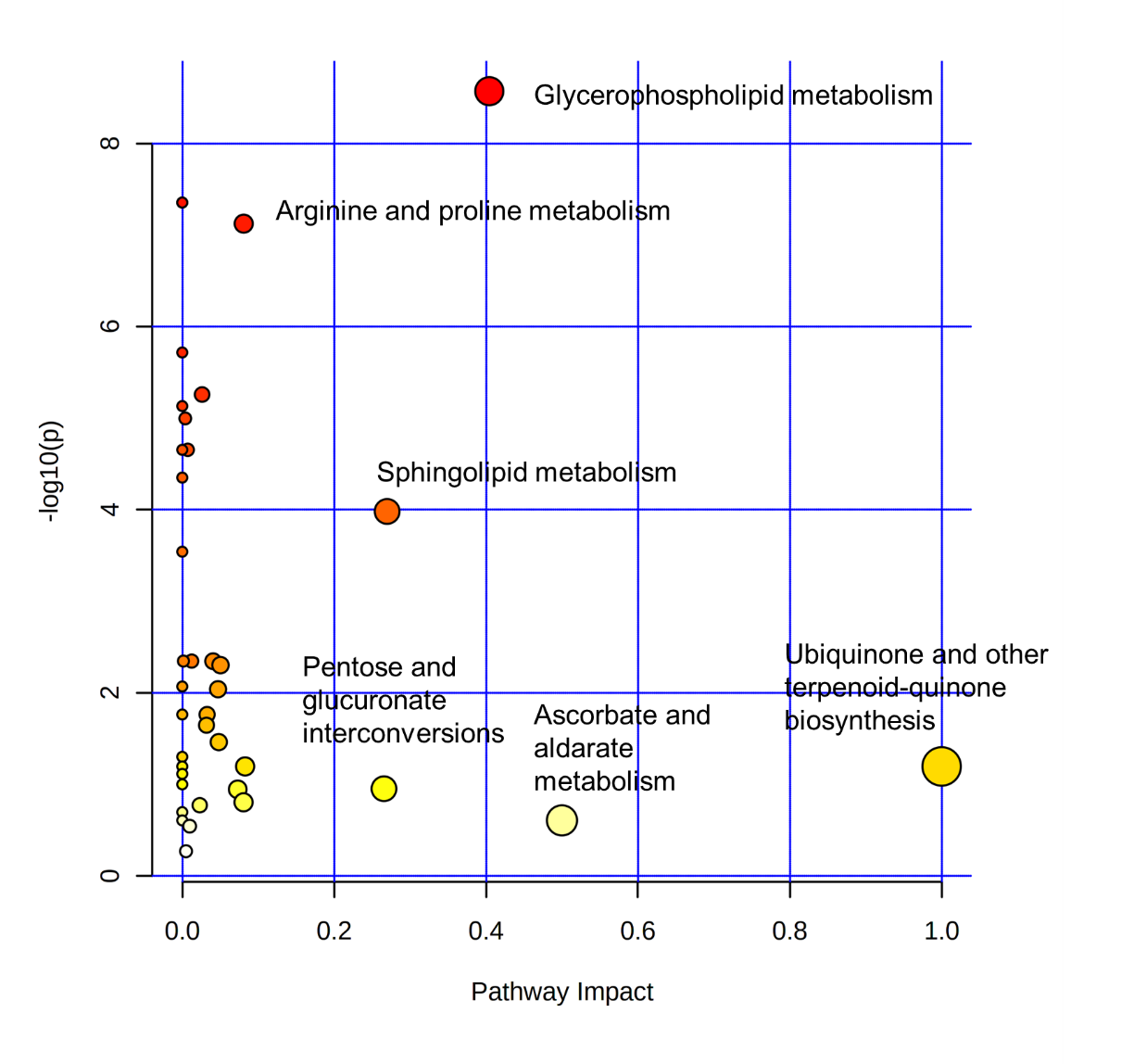


Fig. S2. Heat map of differentiated sphingolipid in each group


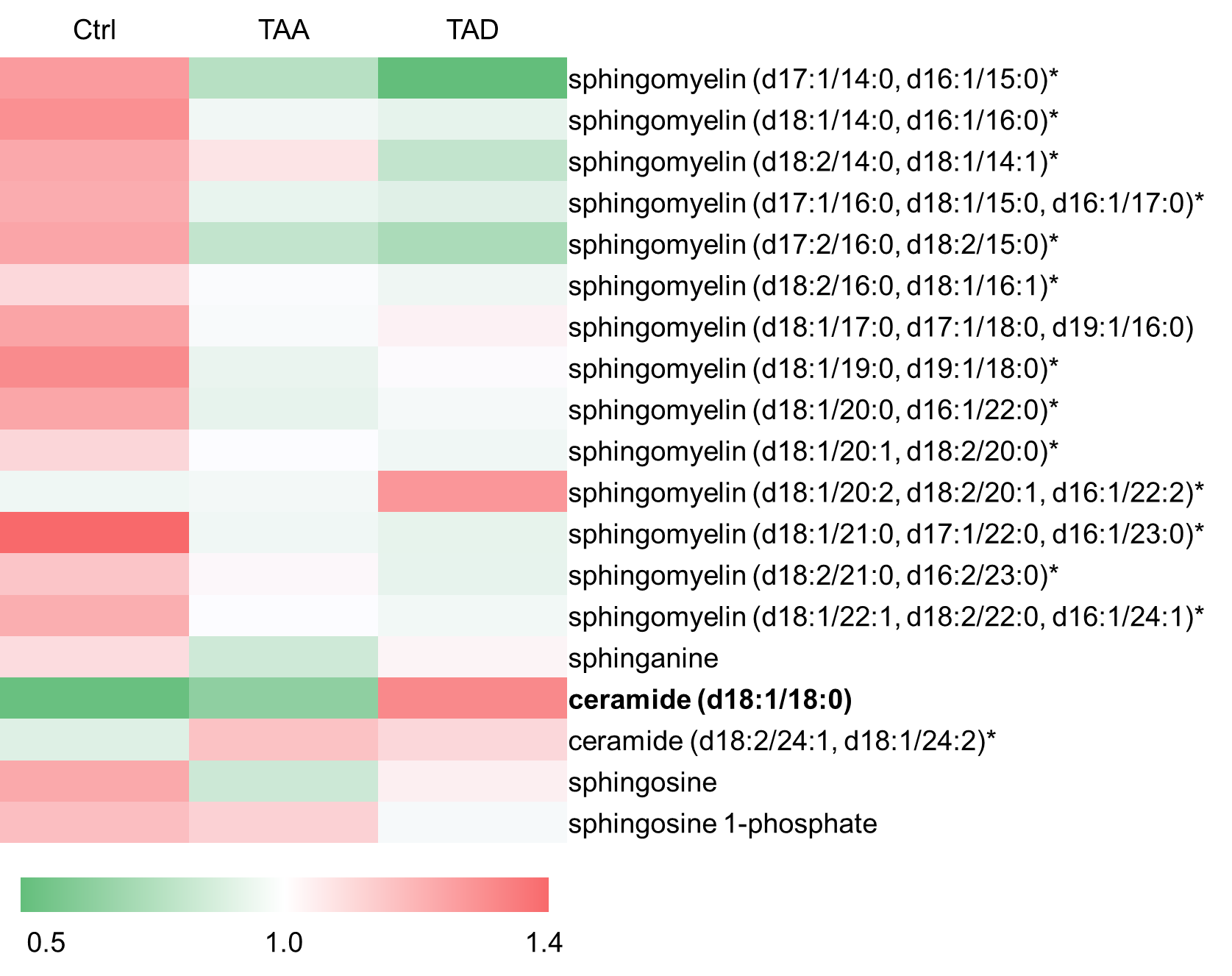


## Supplementary Tables

**Table S1. Primers used in this study**

| **Gene Names** | **Forward primers** | **Reverse primers** |
| --- | --- | --- |
| *Sptlc2* | CGCCTTTGGAAGAGAGATGC | CCGGTGGCGAGAGTACTTTA |
| *Cers6* | TTAGAAGGGCTCTCCAAGCA | TGAAGGTCAGCTGTGAGTGG |
| *Il1b* | GACCTTCCAGGATGAGGACA | AGCTCATATGGGTCCGACAG |
| *Il6* | CCGGAGAGGAGACTTCACAG | TTCTGCAAGTGCATCATCGT |
| *Tnf* | CATCCTGACGCCCTGAAC | TAGTACCGGAGGCATCTTGG |
| *Nlrp3* | ATGGCTGTGTGGATCTTTGC | TATCCCAGCAAACCCATCCA |

**Table S2. Characteristics of the patients included in the discovery cohort**

| **Demographic Information** | **Ctrl (n = 70)** | **TAA (n=70)** | **TAD (n=70)** | ***P* value** |
| --- | --- | --- | --- | --- |
| Male (%) | 43(61.4%) | 44(62.9%) | 45(64.3%) | 0.9408 |
| Age, median (IQR), year-old | 51(44-59.25) | 53(47.75-58) | 52(42.5-62.5) | 0.6783 |
| Hypertension (%) | Not detected | 24(34.3%) | 55 (78.5%) | <0.0001 |
| Hyperlipidemia (%) | 27(38.6%) | 33(47.1%) | 46(65.7%) | 0.0046 |
| Diabetes (%) | 1(1.4%) | 0(0%) | 4(5.7%) | 0.0697 |

The differences in gender ratio, and the incidence of hypertension, Hyperlipidemia and Diabetes between normal controls and patients with TAAD were evaluated by the Chi-square test. Besides, the difference of age in each group was evaluated by the One way analysis of variance (ANOVA).

**Table S3. All differentiated metabolites between TAA and TAD**

| **Biochemical Name** | **KEGG** | **HMDB** | **TAA / Ctrl** | **TAD / Ctrl** | **TAD / TAA** | **Super Pathway** | **Sub Pathway** |
| --- | --- | --- | --- | --- | --- | --- | --- |
|  |  |  |  |  |  |  |  |
|  |  |  |  |  |  |  |  |
| N-acetylglycine |  | [HMDB00532](http://www.hmdb.ca/metabolites/HMDB00532) | 1.02 | **1.36** | **1.33** | Amino Acid | Glycine, Serine and Threonine Metabolism |
| betaine | [C00719](http://www.genome.jp/dbget-bin/www_bget?cpd+C00719) | [HMDB00043](http://www.hmdb.ca/metabolites/HMDB00043) | 0.97 | **0.86** | **0.89** | Amino Acid | Glycine, Serine and Threonine Metabolism |
| N-acetylserine |  | [HMDB02931](http://www.hmdb.ca/metabolites/HMDB02931) | 1.15 | **1.47** | **1.28** | Amino Acid | Glycine, Serine and Threonine Metabolism |
| N-acetylalanine | [C02847](http://www.genome.jp/dbget-bin/www_bget?cpd+C02847) | [HMDB00766](http://www.hmdb.ca/metabolites/HMDB00766) | 1.04 | **1.24** | **1.20** | Amino Acid | Alanine and Aspartate Metabolism |
| glutamate | [C00025](http://www.genome.jp/dbget-bin/www_bget?cpd+C00025) | [HMDB00148](http://www.hmdb.ca/metabolites/HMDB00148) | 0.95 | **0.84** | **0.88** | Amino Acid | Glutamate Metabolism |
| N-acetyl-aspartyl-glutamate (NAAG) | [C12270](http://www.genome.jp/dbget-bin/www_bget?cpd+C12270) | [HMDB01067](http://www.hmdb.ca/metabolites/HMDB01067) | 1.36 | **2.51** | **1.85** | Amino Acid | Glutamate Metabolism |
| beta-citrylglutamate | [C20775](http://www.genome.jp/dbget-bin/www_bget?cpd+C20775) |  | **1.19** | **1.74** | **1.47** | Amino Acid | Glutamate Metabolism |
| 3-methylhistidine | [C01152](http://www.genome.jp/dbget-bin/www_bget?cpd+C01152) | [HMDB00479](http://www.hmdb.ca/metabolites/HMDB00479) | **0.56** | **0.38** | **0.67** | Amino Acid | Histidine Metabolism |
| hydantoin-5-propionate | [C05565](http://www.genome.jp/dbget-bin/www_bget?cpd+C05565) | [HMDB01212](http://www.hmdb.ca/metabolites/HMDB01212) | **5.09** | **9.19** | **1.81** | Amino Acid | Histidine Metabolism |
| imidazole propionate |  | [HMDB02271](http://www.hmdb.ca/metabolites/HMDB02271) | 1.04 | **3.48** | **3.35** | Amino Acid | Histidine Metabolism |
| imidazole lactate | [C05568](http://www.genome.jp/dbget-bin/www_bget?cpd+C05568) | [HMDB02320](http://www.hmdb.ca/metabolites/HMDB02320) | 0.94 | **1.34** | **1.44** | Amino Acid | Histidine Metabolism |
| N6-acetyllysine | [C02727](http://www.genome.jp/dbget-bin/www_bget?cpd+C02727) | [HMDB00206](http://www.hmdb.ca/metabolites/HMDB00206) | **1.12** | **1.35** | **1.21** | Amino Acid | Lysine Metabolism |
| N6,N6,N6-trimethyllysine | [C03793](http://www.genome.jp/dbget-bin/www_bget?cpd+C03793) | [HMDB01325](http://www.hmdb.ca/metabolites/HMDB01325) | 0.78 | 1.11 | **1.42** | Amino Acid | Lysine Metabolism |
| 5-hydroxylysine | [C16741](http://www.genome.jp/dbget-bin/www_bget?cpd+C16741) | [HMDB00450](http://www.hmdb.ca/metabolites/HMDB00450) | **1.13** | **1.42** | **1.25** | Amino Acid | Lysine Metabolism |
| 5-(galactosylhydroxy)-L-lysine |  |  | **1.51** | **1.88** | **1.25** | Amino Acid | Lysine Metabolism |
| fructosyllysine |  |  | **0.75** | **1.00** | **1.33** | Amino Acid | Lysine Metabolism |
| pipecolate | [C00408](http://www.genome.jp/dbget-bin/www_bget?cpd+C00408) | [HMDB00070](http://www.hmdb.ca/metabolites/HMDB00070) | 1.24 | **0.83** | **0.68** | Amino Acid | Lysine Metabolism |
| 6-oxopiperidine-2-carboxylate |  | [HMDB61705](http://www.hmdb.ca/metabolites/HMDB61705) | 1.23 | **2.15** | **1.75** | Amino Acid | Lysine Metabolism |
| phenylalanine | [C00079](http://www.genome.jp/dbget-bin/www_bget?cpd+C00079) | [HMDB00159](http://www.hmdb.ca/metabolites/HMDB00159) | 0.99 | **1.20** | **1.22** | Amino Acid | Phenylalanine Metabolism |
| N-acetylphenylalanine | [C03519](http://www.genome.jp/dbget-bin/www_bget?cpd+C03519) | [HMDB00512](http://www.hmdb.ca/metabolites/HMDB00512) | 0.94 | **1.39** | **1.48** | Amino Acid | Phenylalanine Metabolism |
| 1-carboxyethylphenylalanine |  |  | 1.23 | **3.74** | **3.05** | Amino Acid | Phenylalanine Metabolism |
| phenyllactate (PLA) | [C05607](http://www.genome.jp/dbget-bin/www_bget?cpd+C05607) | [HMDB00779](http://www.hmdb.ca/metabolites/HMDB00779) | 0.99 | **1.88** | **1.90** | Amino Acid | Phenylalanine Metabolism |
| 4-hydroxyphenylpyruvate | [C01179](http://www.genome.jp/dbget-bin/www_bget?cpd+C01179) | [HMDB00707](http://www.hmdb.ca/metabolites/HMDB00707) | 0.98 | **1.36** | **1.39** | Amino Acid | Tyrosine Metabolism |
| 3-(4-hydroxyphenyl)lactate | [C03672](http://www.genome.jp/dbget-bin/www_bget?cpd+C03672) | [HMDB00755](http://www.hmdb.ca/metabolites/HMDB00755) | 1.04 | **1.75** | **1.68** | Amino Acid | Tyrosine Metabolism |
| dopamine 4-sulfate | [C13691](http://www.genome.jp/dbget-bin/www_bget?cpd+C13691) | [HMDB04148](http://www.hmdb.ca/metabolites/HMDB04148) | **2.75** | 1.30 | **0.47** | Amino Acid | Tyrosine Metabolism |
| dopamine 3-O-sulfate | [C13690](http://www.genome.jp/dbget-bin/www_bget?cpd+C13690) | [HMDB06275](http://www.hmdb.ca/metabolites/HMDB06275) | **1.69** | 1.19 | **0.70** | Amino Acid | Tyrosine Metabolism |
| p-cresol glucuronide* |  | [HMDB11686](http://www.hmdb.ca/metabolites/HMDB11686) | 0.86 | **1.56** | **1.83** | Amino Acid | Tyrosine Metabolism |
| thyroxine | [C01829](http://www.genome.jp/dbget-bin/www_bget?cpd+C01829) | [HMDB01918](http://www.hmdb.ca/metabolites/HMDB01918) | **0.83** | **0.69** | **0.83** | Amino Acid | Tyrosine Metabolism |
| C-glycosyltryptophan |  |  | **1.46** | **1.61** | **1.11** | Amino Acid | Tryptophan Metabolism |
| N-formylanthranilic acid | [C05653](http://www.genome.jp/dbget-bin/www_bget?cpd+C05653) | [HMDB04089](http://www.hmdb.ca/metabolites/HMDB04089) | 1.00 | **1.47** | **1.47** | Amino Acid | Tryptophan Metabolism |
| indoleacetate | [C00954](http://www.genome.jp/dbget-bin/www_bget?cpd+C00954) | [HMDB00197](http://www.hmdb.ca/metabolites/HMDB00197) | **1.04** | **0.75** | **0.72** | Amino Acid | Tryptophan Metabolism |
| 6-bromotryptophan |  |  | **0.86** | **0.59** | **0.69** | Amino Acid | Tryptophan Metabolism |
| leucine |  |  | **0.88** | 1.00 | **1.13** | Amino Acid | Leucine, Isoleucine and Valine Metabolism |
| 1-carboxyethylleucine |  |  | 0.97 | **4.04** | **4.18** | Amino Acid | Leucine, Isoleucine and Valine Metabolism |
| alpha-hydroxyisocaproate | [C03264](http://www.genome.jp/dbget-bin/www_bget?cpd+C03264) | [HMDB00746](http://www.hmdb.ca/metabolites/HMDB00746) | 1.03 | **1.47** | **1.43** | Amino Acid | Leucine, Isoleucine and Valine Metabolism |
| 2-ketocaprylate |  | [HMDB13211](http://www.hmdb.ca/metabolites/HMDB13211) | **0.51** | **0.27** | **0.53** | Amino Acid | Leucine, Isoleucine and Valine Metabolism |
| isovalerate (i5:0) | [C08262](http://www.genome.jp/dbget-bin/www_bget?cpd+C08262) | [HMDB00718](http://www.hmdb.ca/metabolites/HMDB00718) | **0.75** | 0.90 | **1.20** | Amino Acid | Leucine, Isoleucine and Valine Metabolism |
| isovalerylcarnitine (C5) |  | [HMDB00688](http://www.hmdb.ca/metabolites/HMDB00688) | **0.52** | **1.16** | **2.21** | Amino Acid | Leucine, Isoleucine and Valine Metabolism |
| beta-hydroxyisovalerate |  | [HMDB00754](http://www.hmdb.ca/metabolites/HMDB00754) | 1.10 | **1.66** | **1.51** | Amino Acid | Leucine, Isoleucine and Valine Metabolism |
| isoleucine | [C00407](http://www.genome.jp/dbget-bin/www_bget?cpd+C00407) | [HMDB00172](http://www.hmdb.ca/metabolites/HMDB00172) | **0.87** | 0.98 | **1.13** | Amino Acid | Leucine, Isoleucine and Valine Metabolism |
| 2-hydroxy-3-methylvalerate |  | [HMDB00317](http://www.hmdb.ca/metabolites/HMDB00317) | 0.79 | **1.34** | **1.69** | Amino Acid | Leucine, Isoleucine and Valine Metabolism |
| 2-methylbutyrylcarnitine (C5) |  | [HMDB00378](http://www.hmdb.ca/metabolites/HMDB00378) | 0.84 | **1.79** | **2.15** | Amino Acid | Leucine, Isoleucine and Valine Metabolism |
| tiglylcarnitine (C5:1-DC) |  | [HMDB02366](http://www.hmdb.ca/metabolites/HMDB02366) | **0.85** | 1.68 | **1.96** | Amino Acid | Leucine, Isoleucine and Valine Metabolism |
| alpha-hydroxyisovalerate |  | [HMDB00407](http://www.hmdb.ca/metabolites/HMDB00407) | 0.99 | **1.57** | **1.59** | Amino Acid | Leucine, Isoleucine and Valine Metabolism |
| 3-hydroxyisobutyrate | [C06001](http://www.genome.jp/dbget-bin/www_bget?cpd+C06001) | [HMDB00336](http://www.hmdb.ca/metabolites/HMDB00336) | 1.01 | **1.57** | **1.55** | Amino Acid | Leucine, Isoleucine and Valine Metabolism |
| methionine | [C00073](http://www.genome.jp/dbget-bin/www_bget?cpd+C00073) | [HMDB00696](http://www.hmdb.ca/metabolites/HMDB00696) | **0.88** | 1.03 | **1.17** | Amino Acid | Methionine, Cysteine, SAM and Taurine Metabolism |
| N-acetylmethionine | [C02712](http://www.genome.jp/dbget-bin/www_bget?cpd+C02712) | [HMDB11745](http://www.hmdb.ca/metabolites/HMDB11745) | **1.40** | **2.30** | **1.64** | Amino Acid | Methionine, Cysteine, SAM and Taurine Metabolism |
| N-formylmethionine | [C03145](http://www.genome.jp/dbget-bin/www_bget?cpd+C03145) | [HMDB01015](http://www.hmdb.ca/metabolites/HMDB01015) | 1.05 | **1.25** | **1.18** | Amino Acid | Methionine, Cysteine, SAM and Taurine Metabolism |
| S-methylmethionine | [C05319](http://www.genome.jp/dbget-bin/www_bget?cpd+C05319) | [HMDB38670](http://www.hmdb.ca/metabolites/HMDB38670) | 1.16 | **0.34** | **0.29** | Amino Acid | Methionine, Cysteine, SAM and Taurine Metabolism |
| 2-hydroxy-4-(methylthio)butanoic acid |  |  | 1.07 | **2.06** | **1.93** | Amino Acid | Methionine, Cysteine, SAM and Taurine Metabolism |
| alpha-ketobutyrate | [C00109](http://www.genome.jp/dbget-bin/www_bget?cpd+C00109) | [HMDB00005](http://www.hmdb.ca/metabolites/HMDB00005) | **0.68** | 1.38 | **2.02** | Amino Acid | Methionine, Cysteine, SAM and Taurine Metabolism |
| S-methylcysteine |  | [HMDB02108](http://www.hmdb.ca/metabolites/HMDB02108) | **0.82** | **0.60** | **0.73** | Amino Acid | Methionine, Cysteine, SAM and Taurine Metabolism |
| S-methylcysteine sulfoxide |  | [HMDB29432](http://www.hmdb.ca/metabolites/HMDB29432) | **0.67** | **0.42** | **0.63** | Amino Acid | Methionine, Cysteine, SAM and Taurine Metabolism |
| cysteine s-sulfate | [C05824](http://www.genome.jp/dbget-bin/www_bget?cpd+C05824) | [HMDB00731](http://www.hmdb.ca/metabolites/HMDB00731) | **0.24** | **0.34** | **1.40** | Amino Acid | Methionine, Cysteine, SAM and Taurine Metabolism |
| taurine | [C00245](http://www.genome.jp/dbget-bin/www_bget?cpd+C00245) | [HMDB00251](http://www.hmdb.ca/metabolites/HMDB00251) | **0.84** | **0.79** | **0.94** | Amino Acid | Methionine, Cysteine, SAM and Taurine Metabolism |
| urea | [C00086](http://www.genome.jp/dbget-bin/www_bget?cpd+C00086) | [HMDB00294](http://www.hmdb.ca/metabolites/HMDB00294) | 1.28 | **1.69** | **1.32** | Amino Acid | Urea cycle; Arginine and Proline Metabolism |
| 2-oxoarginine* | [C03771](http://www.genome.jp/dbget-bin/www_bget?cpd+C03771) | [HMDB04225](http://www.hmdb.ca/metabolites/HMDB04225) | **0.69** | **0.53** | **0.76** | Amino Acid | Urea cycle; Arginine and Proline Metabolism |
| citrulline | [C00327](http://www.genome.jp/dbget-bin/www_bget?cpd+C00327) | [HMDB00904](http://www.hmdb.ca/metabolites/HMDB00904) | 0.97 | **0.76** | **0.78** | Amino Acid | Urea cycle; Arginine and Proline Metabolism |
| homoarginine | [C01924](http://www.genome.jp/dbget-bin/www_bget?cpd+C01924) | [HMDB00670](http://www.hmdb.ca/metabolites/HMDB00670) | **0.76** | **0.64** | **0.84** | Amino Acid | Urea cycle; Arginine and Proline Metabolism |
| N-acetylarginine | [C02562](http://www.genome.jp/dbget-bin/www_bget?cpd+C02562) | [HMDB04620](http://www.hmdb.ca/metabolites/HMDB04620) | 0.89 | **0.72** | **0.81** | Amino Acid | Urea cycle; Arginine and Proline Metabolism |
| N-acetylproline |  |  | **0.81** | **0.59** | **0.73** | Amino Acid | Urea cycle; Arginine and Proline Metabolism |
| N-delta-acetylornithine |  |  | 0.85 | **0.65** | **0.76** | Amino Acid | Urea cycle; Arginine and Proline Metabolism |
| pro-hydroxy-pro |  | [HMDB06695](http://www.hmdb.ca/metabolites/HMDB06695) | **1.34** | **1.99** | **1.48** | Amino Acid | Urea cycle; Arginine and Proline Metabolism |
| N,N,N-trimethyl-alanylproline betaine (TMAP) |  |  | 1.42 | **3.18** | **2.23** | Amino Acid | Urea cycle; Arginine and Proline Metabolism |
| argininate* |  | [HMDB03148](http://www.hmdb.ca/metabolites/HMDB03148) | **0.76** | **0.69** | **0.90** | Amino Acid | Urea cycle; Arginine and Proline Metabolism |
| guanidinoacetate | [C00581](http://www.genome.jp/dbget-bin/www_bget?cpd+C00581) | [HMDB00128](http://www.hmdb.ca/metabolites/HMDB00128) | 0.87 | **0.53** | **0.61** | Amino Acid | Creatine Metabolism |
| N-acetylputrescine | [C02714](http://www.genome.jp/dbget-bin/www_bget?cpd+C02714) | [HMDB02064](http://www.hmdb.ca/metabolites/HMDB02064) | **1.17** | **1.64** | **1.41** | Amino Acid | Polyamine Metabolism |
| spermidine | [C00315](http://www.genome.jp/dbget-bin/www_bget?cpd+C00315) | [HMDB01257](http://www.hmdb.ca/metabolites/HMDB01257) | **0.78** | 1.47 | **1.88** | Amino Acid | Polyamine Metabolism |
| (N(1) + N(8))-acetylspermidine |  |  | **1.42** | **3.62** | **2.55** | Amino Acid | Polyamine Metabolism |
| 5-methylthioadenosine (MTA) | [C00170](http://www.genome.jp/dbget-bin/www_bget?cpd+C00170) | [HMDB01173](http://www.hmdb.ca/metabolites/HMDB01173) | 1.11 | **2.83** | **2.56** | Amino Acid | Polyamine Metabolism |
| 2-aminobutyrate | [C02261](http://www.genome.jp/dbget-bin/www_bget?cpd+C02261) | [HMDB00650](http://www.hmdb.ca/metabolites/HMDB00650) | **0.81** | 1.07 | **1.33** | Amino Acid | Glutathione Metabolism |
| 2-hydroxybutyrate/2-hydroxyisobutyrate |  |  | **1.33** | **2.41** | **1.81** | Amino Acid | Glutathione Metabolism |
| gamma-glutamylglutamate | [C05282](http://www.genome.jp/dbget-bin/www_bget?cpd+C05282) | [HMDB11737](http://www.hmdb.ca/metabolites/HMDB11737) | **1.27** | 1.07 | **0.84** | Peptide | Gamma-glutamyl Amino Acid |
| gamma-glutamylglycine |  | [HMDB11667](http://www.hmdb.ca/metabolites/HMDB11667) | **0.85** | **0.74** | **0.86** | Peptide | Gamma-glutamyl Amino Acid |
| gamma-glutamylhistidine |  |  | **0.89** | **0.76** | **0.85** | Peptide | Gamma-glutamyl Amino Acid |
| gamma-glutamylleucine |  | [HMDB11171](http://www.hmdb.ca/metabolites/HMDB11171) | **0.80** | 0.98 | **1.21** | Peptide | Gamma-glutamyl Amino Acid |
| gamma-glutamylphenylalanine |  | [HMDB00594](http://www.hmdb.ca/metabolites/HMDB00594) | 0.97 | **1.43** | **1.47** | Peptide | Gamma-glutamyl Amino Acid |
| gamma-glutamylthreonine |  | [HMDB29159](http://www.hmdb.ca/metabolites/HMDB29159) | **0.89** | **0.80** | **0.90** | Peptide | Gamma-glutamyl Amino Acid |
| gamma-glutamylcitrulline* |  |  | **0.80** | **0.54** | **0.67** | Peptide | Gamma-glutamyl Amino Acid |
| isoleucylglycine |  | [HMDB28907](http://www.hmdb.ca/metabolites/HMDB28907) | 1.64 | **3.11** | **1.90** | Peptide | Dipeptide |
| leucylglycine |  | [HMDB28929](http://www.hmdb.ca/metabolites/HMDB28929) | 1.05 | **1.53** | **1.46** | Peptide | Dipeptide |
| phenylacetylglutamine | [C04148](http://www.genome.jp/dbget-bin/www_bget?cpd+C04148) | [HMDB06344](http://www.hmdb.ca/metabolites/HMDB06344) | 1.16 | **2.13** | **1.83** | Peptide | Acetylated Peptides |
| 4-hydroxyphenylacetylglutamine |  |  | 2.87 | **8.80** | **3.07** | Peptide | Acetylated Peptides |
| 1,5-anhydroglucitol (1,5-AG) | [C07326](http://www.genome.jp/dbget-bin/www_bget?cpd+C07326) | [HMDB02712](http://www.hmdb.ca/metabolites/HMDB02712) | 1.21 | **0.76** | **0.63** | Carbohydrate | Glycolysis, Gluconeogenesis, and Pyruvate Metabolism |
| glucose | [C00031](http://www.genome.jp/dbget-bin/www_bget?cpd+C00031) | [HMDB00122](http://www.hmdb.ca/metabolites/HMDB00122) | 1.03 | **1.20** | **1.16** | Carbohydrate | Glycolysis, Gluconeogenesis, and Pyruvate Metabolism |
| pyruvate | [C00022](http://www.genome.jp/dbget-bin/www_bget?cpd+C00022) | [HMDB00243](http://www.hmdb.ca/metabolites/HMDB00243) | **0.52** | **0.68** | **1.30** | Carbohydrate | Glycolysis, Gluconeogenesis, and Pyruvate Metabolism |
| lactate | [C00186](http://www.genome.jp/dbget-bin/www_bget?cpd+C00186) | [HMDB00190](http://www.hmdb.ca/metabolites/HMDB00190) | **0.82** | **0.95** | **1.16** | Carbohydrate | Glycolysis, Gluconeogenesis, and Pyruvate Metabolism |
| glycerate | [C00258](http://www.genome.jp/dbget-bin/www_bget?cpd+C00258) | [HMDB00139](http://www.hmdb.ca/metabolites/HMDB00139) | **0.73** | **0.71** | **0.97** | Carbohydrate | Glycolysis, Gluconeogenesis, and Pyruvate Metabolism |
| ribulonate/xylulonate/lyxonate* |  |  | **0.77** | **0.70** | **0.91** | Carbohydrate | Pentose Metabolism |
| maltotriose | [C01835](http://www.genome.jp/dbget-bin/www_bget?cpd+C01835) | [HMDB01262](http://www.hmdb.ca/metabolites/HMDB01262) | 1.71 | **18.08** | **10.59** | Carbohydrate | Glycogen Metabolism |
| maltose | [C00208](http://www.genome.jp/dbget-bin/www_bget?cpd+C00208) | [HMDB00163](http://www.hmdb.ca/metabolites/HMDB00163) | 2.12 | **7.57** | **3.56** | Carbohydrate | Glycogen Metabolism |
| mannose | [C00159](http://www.genome.jp/dbget-bin/www_bget?cpd+C00159) | [HMDB00169](http://www.hmdb.ca/metabolites/HMDB00169) | 1.04 | **2.10** | **2.02** | Carbohydrate | Fructose, Mannose and Galactose Metabolism |
| glucuronate | [C00191](http://www.genome.jp/dbget-bin/www_bget?cpd+C00191) | [HMDB00127](http://www.hmdb.ca/metabolites/HMDB00127) | 1.28 | 2.04 | **1.60** | Carbohydrate | Aminosugar Metabolism |
| N-acetylneuraminate | [C00270](http://www.genome.jp/dbget-bin/www_bget?cpd+C00270) | [HMDB00230](http://www.hmdb.ca/metabolites/HMDB00230) | **1.41** | **2.00** | **1.41** | Carbohydrate | Aminosugar Metabolism |
| N-acetylglucosamine/N-acetylgalactosamine |  | [HMDB00215](http://www.hmdb.ca/metabolites/HMDB00215) | **1.23** | **1.65** | **1.34** | Carbohydrate | Aminosugar Metabolism |
| citrate | [C00158](http://www.genome.jp/dbget-bin/www_bget?cpd+C00158) | [HMDB00094](http://www.hmdb.ca/metabolites/HMDB00094) | 1.04 | **0.93** | **0.90** | Energy | TCA Cycle |
| aconitate [cis or trans] |  |  | **0.88** | **0.77** | **0.88** | Energy | TCA Cycle |
| succinate | [C00042](http://www.genome.jp/dbget-bin/www_bget?cpd+C00042) | [HMDB00254](http://www.hmdb.ca/metabolites/HMDB00254) | 0.98 | **2.08** | **2.12** | Energy | TCA Cycle |
| 2-methylcitrate/homocitrate |  |  | 1.80 | **2.55** | **1.42** | Energy | TCA Cycle |
| malonylcarnitine |  | [HMDB02095](http://www.hmdb.ca/metabolites/HMDB02095) | 1.23 | **1.49** | **1.21** | Lipid | Fatty Acid Synthesis |
| caproate (6:0) | [C01585](http://www.genome.jp/dbget-bin/www_bget?cpd+C01585) | [HMDB00535](http://www.hmdb.ca/metabolites/HMDB00535) | **0.70** | **0.63** | **0.90** | Lipid | Medium Chain Fatty Acid |
| heptanoate (7:0) | [C17714](http://www.genome.jp/dbget-bin/www_bget?cpd+C17714) | [HMDB00666](http://www.hmdb.ca/metabolites/HMDB00666) | 1.03 | **0.79** | **0.77** | Lipid | Medium Chain Fatty Acid |
| pelargonate (9:0) | [C01601](http://www.genome.jp/dbget-bin/www_bget?cpd+C01601) | [HMDB00847](http://www.hmdb.ca/metabolites/HMDB00847) | 1.01 | **0.82** | **0.82** | Lipid | Medium Chain Fatty Acid |
| cis-4-decenoate (10:1n6)* |  |  | **0.54** | **0.42** | **0.79** | Lipid | Medium Chain Fatty Acid |
| (2 or 3)-decenoate (10:1n7 or n8) |  |  | **0.58** | **0.49** | **0.85** | Lipid | Medium Chain Fatty Acid |
| undecanoate (11:0) | [C17715](http://www.genome.jp/dbget-bin/www_bget?cpd+C17715) | [HMDB00947](http://www.hmdb.ca/metabolites/HMDB00947) | 1.06 | 0.99 | **0.94** | Lipid | Medium Chain Fatty Acid |
| 10-undecenoate (11:1n1) | [C13910](http://www.genome.jp/dbget-bin/www_bget?cpd+C13910) | [HMDB33724](http://www.hmdb.ca/metabolites/HMDB33724) | **0.56** | **0.42** | **0.75** | Lipid | Medium Chain Fatty Acid |
| laurate (12:0) | [C02679](http://www.genome.jp/dbget-bin/www_bget?cpd+C02679) | [HMDB00638](http://www.hmdb.ca/metabolites/HMDB00638) | 0.99 | **0.86** | **0.87** | Lipid | Medium Chain Fatty Acid |
| 5-dodecenoate (12:1n7) |  | [HMDB00529](http://www.hmdb.ca/metabolites/HMDB00529) | **0.72** | **0.55** | **0.76** | Lipid | Medium Chain Fatty Acid |
| myristate (14:0) | [C06424](http://www.genome.jp/dbget-bin/www_bget?cpd+C06424) | [HMDB00806](http://www.hmdb.ca/metabolites/HMDB00806) | 1.12 | **0.90** | **0.81** | Lipid | Long Chain Saturated Fatty Acid |
| pentadecanoate (15:0) | [C16537](http://www.genome.jp/dbget-bin/www_bget?cpd+C16537) | [HMDB00826](http://www.hmdb.ca/metabolites/HMDB00826) | 1.04 | **0.90** | **0.87** | Lipid | Long Chain Saturated Fatty Acid |
| palmitate (16:0) | [C00249](http://www.genome.jp/dbget-bin/www_bget?cpd+C00249) | [HMDB00220](http://www.hmdb.ca/metabolites/HMDB00220) | 1.10 | 0.95 | **0.86** | Lipid | Long Chain Saturated Fatty Acid |
| margarate (17:0) |  | [HMDB02259](http://www.hmdb.ca/metabolites/HMDB02259) | 1.12 | **0.88** | **0.78** | Lipid | Long Chain Saturated Fatty Acid |
| stearate (18:0) | [C01530](http://www.genome.jp/dbget-bin/www_bget?cpd+C01530) | [HMDB00827](http://www.hmdb.ca/metabolites/HMDB00827) | **1.13** | 1.00 | **0.89** | Lipid | Long Chain Saturated Fatty Acid |
| nonadecanoate (19:0) | [C16535](http://www.genome.jp/dbget-bin/www_bget?cpd+C16535) | [HMDB00772](http://www.hmdb.ca/metabolites/HMDB00772) | 1.09 | 0.92 | **0.85** | Lipid | Long Chain Saturated Fatty Acid |
| myristoleate (14:1n5) | [C08322](http://www.genome.jp/dbget-bin/www_bget?cpd+C08322) | [HMDB02000](http://www.hmdb.ca/metabolites/HMDB02000) | 1.04 | **0.73** | **0.71** | Lipid | Long Chain Monounsaturated Fatty Acid |
| palmitoleate (16:1n7) | [C08362](http://www.genome.jp/dbget-bin/www_bget?cpd+C08362) | [HMDB03229](http://www.hmdb.ca/metabolites/HMDB03229) | **1.35** | 0.97 | **0.72** | Lipid | Long Chain Monounsaturated Fatty Acid |
| 10-heptadecenoate (17:1n7) |  | [HMDB60038](http://www.hmdb.ca/metabolites/HMDB60038) | 1.19 | 0.86 | **0.72** | Lipid | Long Chain Monounsaturated Fatty Acid |
| 10-nonadecenoate (19:1n9) |  | [HMDB13622](http://www.hmdb.ca/metabolites/HMDB13622) | **1.29** | 0.96 | **0.75** | Lipid | Long Chain Monounsaturated Fatty Acid |
| erucate (22:1n9) | [C08316](http://www.genome.jp/dbget-bin/www_bget?cpd+C08316) | [HMDB02068](http://www.hmdb.ca/metabolites/HMDB02068) | **2.09** | 0.86 | **0.41** | Lipid | Long Chain Monounsaturated Fatty Acid |
| tetradecadienoate (14:2)* |  | [HMDB00560](http://www.hmdb.ca/metabolites/HMDB00560) | **0.69** | **0.52** | **0.75** | Lipid | Long Chain Polyunsaturated Fatty Acid (n3 and n6) |
| stearidonate (18:4n3) | [C16300](http://www.genome.jp/dbget-bin/www_bget?cpd+C16300) | [HMDB06547](http://www.hmdb.ca/metabolites/HMDB06547) | **1.52** | **0.67** | **0.44** | Lipid | Long Chain Polyunsaturated Fatty Acid (n3 and n6) |
| eicosapentaenoate (EPA; 20:5n3) | [C06428](http://www.genome.jp/dbget-bin/www_bget?cpd+C06428) | [HMDB01999](http://www.hmdb.ca/metabolites/HMDB01999) | **0.74** | **0.53** | **0.72** | Lipid | Long Chain Polyunsaturated Fatty Acid (n3 and n6) |
| docosapentaenoate (n3 DPA; 22:5n3) | [C16513](http://www.genome.jp/dbget-bin/www_bget?cpd+C16513) | [HMDB06528](http://www.hmdb.ca/metabolites/HMDB06528) | 1.26 | **0.76** | **0.60** | Lipid | Long Chain Polyunsaturated Fatty Acid (n3 and n6) |
| docosahexaenoate (DHA; 22:6n3) | [C06429](http://www.genome.jp/dbget-bin/www_bget?cpd+C06429) | [HMDB02183](http://www.hmdb.ca/metabolites/HMDB02183) | **0.75** | **0.55** | **0.73** | Lipid | Long Chain Polyunsaturated Fatty Acid (n3 and n6) |
| docosatrienoate (22:3n3) | [C16534](http://www.genome.jp/dbget-bin/www_bget?cpd+C16534) | [HMDB02823](http://www.hmdb.ca/metabolites/HMDB02823) | **1.50** | 1.12 | **0.75** | Lipid | Long Chain Polyunsaturated Fatty Acid (n3 and n6) |
| nisinate (24:6n3) |  | [HMDB02007](http://www.hmdb.ca/metabolites/HMDB02007) | **0.75** | **0.36** | **0.48** | Lipid | Long Chain Polyunsaturated Fatty Acid (n3 and n6) |
| hexadecadienoate (16:2n6) |  | [HMDB00477](http://www.hmdb.ca/metabolites/HMDB00477) | 1.25 | **0.82** | **0.66** | Lipid | Long Chain Polyunsaturated Fatty Acid (n3 and n6) |
| linoleate (18:2n6) | [C01595](http://www.genome.jp/dbget-bin/www_bget?cpd+C01595) | [HMDB00673](http://www.hmdb.ca/metabolites/HMDB00673) | **1.20** | 0.97 | **0.81** | Lipid | Long Chain Polyunsaturated Fatty Acid (n3 and n6) |
| linolenate [alpha or gamma; (18:3n3 or 6)] | [C06426](http://www.genome.jp/dbget-bin/www_bget?cpd+C06426) | [HMDB03073](http://www.hmdb.ca/metabolites/HMDB03073) | **1.64** | 0.91 | **0.55** | Lipid | Long Chain Polyunsaturated Fatty Acid (n3 and n6) |
| dihomo-linoleate (20:2n6) | [C16525](http://www.genome.jp/dbget-bin/www_bget?cpd+C16525) | [HMDB05060](http://www.hmdb.ca/metabolites/HMDB05060) | **1.34** | 1.05 | **0.78** | Lipid | Long Chain Polyunsaturated Fatty Acid (n3 and n6) |
| dihomo-linolenate (20:3n3 or n6) | [C03242](http://www.genome.jp/dbget-bin/www_bget?cpd+C03242) | [HMDB02925](http://www.hmdb.ca/metabolites/HMDB02925) | **1.31** | **0.86** | **0.66** | Lipid | Long Chain Polyunsaturated Fatty Acid (n3 and n6) |
| arachidonate (20:4n6) | [C00219](http://www.genome.jp/dbget-bin/www_bget?cpd+C00219) | [HMDB01043](http://www.hmdb.ca/metabolites/HMDB01043) | 0.90 | **0.71** | **0.79** | Lipid | Long Chain Polyunsaturated Fatty Acid (n3 and n6) |
| adrenate (22:4n6) | [C16527](http://www.genome.jp/dbget-bin/www_bget?cpd+C16527) | [HMDB02226](http://www.hmdb.ca/metabolites/HMDB02226) | **1.39** | 1.10 | **0.79** | Lipid | Long Chain Polyunsaturated Fatty Acid (n3 and n6) |
| docosapentaenoate (n6 DPA; 22:5n6) | [C16513](http://www.genome.jp/dbget-bin/www_bget?cpd+C16513) | [HMDB01976](http://www.hmdb.ca/metabolites/HMDB01976) | 1.14 | **0.86** | **0.75** | Lipid | Long Chain Polyunsaturated Fatty Acid (n3 and n6) |
| sebacate (C10-DC) | [C08277](http://www.genome.jp/dbget-bin/www_bget?cpd+C08277) | [HMDB00792](http://www.hmdb.ca/metabolites/HMDB00792) | 2.05 | 0.89 | **0.43** | Lipid | Fatty Acid, Dicarboxylate |
| dodecanedioate (C12-DC) | [C02678](http://www.genome.jp/dbget-bin/www_bget?cpd+C02678) | [HMDB00623](http://www.hmdb.ca/metabolites/HMDB00623) | **1.93** | 0.90 | **0.46** | Lipid | Fatty Acid, Dicarboxylate |
| 3-hydroxydodecanedioate* |  | [HMDB00413](http://www.hmdb.ca/metabolites/HMDB00413) | **4.08** | 1.47 | **0.36** | Lipid | Fatty Acid, Dicarboxylate |
| dodecadienoate (12:2)* |  |  | **0.51** | **0.39** | **0.77** | Lipid | Fatty Acid, Dicarboxylate |
| tridecenedioate (C13:1-DC)* |  |  | 1.05 | **0.78** | **0.74** | Lipid | Fatty Acid, Dicarboxylate |
| tetradecanedioate (C14-DC) |  | [HMDB00872](http://www.hmdb.ca/metabolites/HMDB00872) | **2.63** | 1.23 | **0.47** | Lipid | Fatty Acid, Dicarboxylate |
| branched chain 14:0 dicarboxylic acid** |  |  | **0.57** | **0.29** | **0.50** | Lipid | Fatty Acid, Dicarboxylate |
| tetradecadienedioate (C14:2-DC)* |  |  | **2.22** | 1.11 | **0.50** | Lipid | Fatty Acid, Dicarboxylate |
| hexadecanedioate (C16-DC) | [C19615](http://www.genome.jp/dbget-bin/www_bget?cpd+C19615) | [HMDB00672](http://www.hmdb.ca/metabolites/HMDB00672) | **2.33** | **1.53** | **0.66** | Lipid | Fatty Acid, Dicarboxylate |
| hexadecenedioate (C16:1-DC)* |  |  | 1.68 | **0.86** | **0.51** | Lipid | Fatty Acid, Dicarboxylate |
| octadecadienedioate (C18:2-DC)* |  |  | 1.04 | **0.58** | **0.56** | Lipid | Fatty Acid, Dicarboxylate |
| eicosanedioate (C20-DC) |  |  | **0.56** | **0.39** | **0.69** | Lipid | Fatty Acid, Dicarboxylate |
| eicosenedioate (C20:1-DC)* |  |  | **0.81** | **0.62** | **0.76** | Lipid | Fatty Acid, Dicarboxylate |
| docosadioate (C22-DC) | [C19625](http://www.genome.jp/dbget-bin/www_bget?cpd+C19625) |  | **0.65** | **0.38** | **0.59** | Lipid | Fatty Acid, Dicarboxylate |
| 3-carboxy-4-methyl-5-pentyl-2-furanpropionate (3-CMPFP)** |  |  | **0.51** | **0.35** | **0.68** | Lipid | Fatty Acid, Dicarboxylate |
| 2-hydroxysebacate |  | [HMDB00424](http://www.hmdb.ca/metabolites/HMDB00424) | **0.64** | **0.32** | **0.51** | Lipid | Fatty Acid, Dicarboxylate |
| linoleamide (18:2n6) |  |  | **10.90** | **4.35** | **0.40** | Lipid | Fatty Acid, Amide |
| oleamide | [C19670](http://www.genome.jp/dbget-bin/www_bget?cpd+C19670) | [HMDB02117](http://www.hmdb.ca/metabolites/HMDB02117) | **35.81** | **13.47** | **0.38** | Lipid | Fatty Acid, Amide |
| palmitamide (16:0) |  | [HMDB12273](http://www.hmdb.ca/metabolites/HMDB12273) | **5.35** | **2.45** | **0.46** | Lipid | Fatty Acid, Amide |
| 2-aminoheptanoate |  |  | **0.64** | **0.46** | **0.72** | Lipid | Fatty Acid, Amino |
| 2-aminooctanoate |  | [HMDB00991](http://www.hmdb.ca/metabolites/HMDB00991) | **0.57** | **0.26** | **0.46** | Lipid | Fatty Acid, Amino |
| N-acetyl-2-aminooctanoate* |  | [HMDB59745](http://www.hmdb.ca/metabolites/HMDB59745) | **0.54** | **0.24** | **0.44** | Lipid | Fatty Acid, Amino |
| propionylcarnitine (C3) | [C03017](http://www.genome.jp/dbget-bin/www_bget?cpd+C03017) | [HMDB00824](http://www.hmdb.ca/metabolites/HMDB00824) | **0.78** | 1.18 | **1.51** | Lipid | Fatty Acid Metabolism (also BCAA Metabolism) |
| 2-methylmalonylcarnitine (C4-DC) |  | [HMDB13133](http://www.hmdb.ca/metabolites/HMDB13133) | 1.07 | **2.06** | **1.93** | Lipid | Fatty Acid Metabolism (also BCAA Metabolism) |
| hexanoylglutamine |  |  | **1.73** | **2.42** | **1.39** | Lipid | Fatty Acid Metabolism (Acyl Glutamine) |
| N-palmitoylglycine |  | [HMDB13034](http://www.hmdb.ca/metabolites/HMDB13034) | 1.08 | **0.90** | **0.83** | Lipid | Fatty Acid Metabolism (Acyl Glycine) |
| N-linoleoylglycine |  |  | **1.45** | 1.00 | **0.69** | Lipid | Fatty Acid Metabolism (Acyl Glycine) |
| palmitoylcarnitine (C16) | [C02990](http://www.genome.jp/dbget-bin/www_bget?cpd+C02990) | [HMDB00222](http://www.hmdb.ca/metabolites/HMDB00222) | **0.89** | **0.78** | **0.88** | Lipid | Fatty Acid Metabolism (Acyl Carnitine, Long Chain Saturated) |
| stearoylcarnitine (C18) |  | [HMDB00848](http://www.hmdb.ca/metabolites/HMDB00848) | **0.72** | **0.60** | **0.84** | Lipid | Fatty Acid Metabolism (Acyl Carnitine, Long Chain Saturated) |
| lignoceroylcarnitine (C24)* |  |  | **0.50** | **0.57** | **1.14** | Lipid | Fatty Acid Metabolism (Acyl Carnitine, Long Chain Saturated) |
| nervonoylcarnitine (C24:1)* |  |  | **0.60** | **0.43** | **0.71** | Lipid | Fatty Acid Metabolism (Acyl Carnitine, Monounsaturated) |
| ximenoylcarnitine (C26:1)* |  |  | **0.74** | 0.92 | **1.24** | Lipid | Fatty Acid Metabolism (Acyl Carnitine, Monounsaturated) |
| linoleoylcarnitine (C18:2)* |  | [HMDB06469](http://www.hmdb.ca/metabolites/HMDB06469) | **0.84** | **0.72** | **0.86** | Lipid | Fatty Acid Metabolism (Acyl Carnitine, Polyunsaturated) |
| linolenoylcarnitine (C18:3)* |  |  | 0.94 | **0.61** | **0.65** | Lipid | Fatty Acid Metabolism (Acyl Carnitine, Polyunsaturated) |
| dihomo-linoleoylcarnitine (C20:2)* |  |  | **0.85** | **0.64** | **0.75** | Lipid | Fatty Acid Metabolism (Acyl Carnitine, Polyunsaturated) |
| arachidonoylcarnitine (C20:4) |  |  | **0.84** | **0.69** | **0.82** | Lipid | Fatty Acid Metabolism (Acyl Carnitine, Polyunsaturated) |
| dihomo-linolenoylcarnitine (C20:3n3 or 6)* |  |  | 0.97 | **0.72** | **0.74** | Lipid | Fatty Acid Metabolism (Acyl Carnitine, Polyunsaturated) |
| adipoylcarnitine (C6-DC) |  | [HMDB61677](http://www.hmdb.ca/metabolites/HMDB61677) | 1.19 | **2.56** | **2.15** | Lipid | Fatty Acid Metabolism (Acyl Carnitine, Dicarboxylate) |
| suberoylcarnitine (C8-DC) |  |  | **3.01** | **4.32** | **1.44** | Lipid | Fatty Acid Metabolism (Acyl Carnitine, Dicarboxylate) |
| (R)-3-hydroxybutyrylcarnitine |  | [HMDB13127](http://www.hmdb.ca/metabolites/HMDB13127) | **1.64** | **2.95** | **1.80** | Lipid | Fatty Acid Metabolism (Acyl Carnitine, Hydroxy) |
| deoxycarnitine | [C01181](http://www.genome.jp/dbget-bin/www_bget?cpd+C01181) | [HMDB01161](http://www.hmdb.ca/metabolites/HMDB01161) | **0.89** | 1.12 | **1.26** | Lipid | Carnitine Metabolism |
| palmitoylcholine |  |  | 1.09 | **0.70** | **0.64** | Lipid | Fatty Acid Metabolism (Acyl Choline) |
| oleoylcholine |  |  | 1.40 | 0.91 | **0.65** | Lipid | Fatty Acid Metabolism (Acyl Choline) |
| linoleoylcholine* |  |  | 0.94 | **0.57** | **0.61** | Lipid | Fatty Acid Metabolism (Acyl Choline) |
| arachidonoylcholine |  |  | 1.04 | **0.60** | **0.58** | Lipid | Fatty Acid Metabolism (Acyl Choline) |
| 2-hydroxyoctanoate |  | [HMDB02264](http://www.hmdb.ca/metabolites/HMDB02264) | **0.57** | **0.39** | **0.69** | Lipid | Fatty Acid, Monohydroxy |
| 2-hydroxydecanoate |  |  | **0.50** | **0.32** | **0.64** | Lipid | Fatty Acid, Monohydroxy |
| 2-hydroxypalmitate |  | [HMDB31057](http://www.hmdb.ca/metabolites/HMDB31057) | 0.97 | **0.77** | **0.80** | Lipid | Fatty Acid, Monohydroxy |
| 2-hydroxystearate | [C03045](http://www.genome.jp/dbget-bin/www_bget?cpd+C03045) |  | 1.00 | **0.78** | **0.79** | Lipid | Fatty Acid, Monohydroxy |
| 2-hydroxyarachidate* |  |  | 0.92 | **0.74** | **0.81** | Lipid | Fatty Acid, Monohydroxy |
| 2-hydroxybehenate |  |  | 0.95 | **0.69** | **0.72** | Lipid | Fatty Acid, Monohydroxy |
| 3-hydroxydecanoate |  | [HMDB02203](http://www.hmdb.ca/metabolites/HMDB02203) | **0.72** | **0.58** | **0.80** | Lipid | Fatty Acid, Monohydroxy |
| 16-hydroxypalmitate | [C18218](http://www.genome.jp/dbget-bin/www_bget?cpd+C18218) | [HMDB06294](http://www.hmdb.ca/metabolites/HMDB06294) | **1.34** | 0.99 | **0.74** | Lipid | Fatty Acid, Monohydroxy |
| 13-HODE + 9-HODE |  |  | **1.47** | 1.14 | **0.77** | Lipid | Fatty Acid, Monohydroxy |
| 9-hydroxystearate |  | [HMDB61661](http://www.hmdb.ca/metabolites/HMDB61661) | **1.80** | 1.14 | **0.63** | Lipid | Fatty Acid, Monohydroxy |
| 12,13-DiHOME | [C14829](http://www.genome.jp/dbget-bin/www_bget?cpd+C14829) | [HMDB04705](http://www.hmdb.ca/metabolites/HMDB04705) | **1.92** | 1.08 | **0.57** | Lipid | Fatty Acid, Dihydroxy |
| 9,10-DiHOME | [C14828](http://www.genome.jp/dbget-bin/www_bget?cpd+C14828) | [HMDB04704](http://www.hmdb.ca/metabolites/HMDB04704) | 1.18 | **0.70** | **0.60** | Lipid | Fatty Acid, Dihydroxy |
| 2S,3R-dihydroxybutyrate |  | [HMDB02453](http://www.hmdb.ca/metabolites/HMDB02453) | 1.07 | **2.30** | **2.15** | Lipid | Fatty Acid, Dihydroxy |
| palmitoyl ethanolamide | [C16512](http://www.genome.jp/dbget-bin/www_bget?cpd+C16512) | [HMDB02100](http://www.hmdb.ca/metabolites/HMDB02100) | **1.45** | **1.24** | **0.86** | Lipid | Endocannabinoid |
| linoleoyl ethanolamide |  | [HMDB12252](http://www.hmdb.ca/metabolites/HMDB12252) | **2.16** | **1.70** | **0.79** | Lipid | Endocannabinoid |
| N-oleoylserine |  |  | **1.18** | 0.98 | **0.83** | Lipid | Endocannabinoid |
| glycerophosphorylcholine (GPC) | [C00670](http://www.genome.jp/dbget-bin/www_bget?cpd+C00670) | [HMDB00086](http://www.hmdb.ca/metabolites/HMDB00086) | **0.58** | **0.40** | **0.69** | Lipid | Phospholipid Metabolism |
| glycerophosphoethanolamine | [C01233](http://www.genome.jp/dbget-bin/www_bget?cpd+C01233) | [HMDB00114](http://www.hmdb.ca/metabolites/HMDB00114) | **0.71** | **0.56** | **0.79** | Lipid | Phospholipid Metabolism |
| 1-myristoyl-2-palmitoyl-GPC (14:0/16:0) |  | [HMDB07869](http://www.hmdb.ca/metabolites/HMDB07869) | **1.41** | **0.86** | **0.61** | Lipid | Phosphatidylcholine (PC) |
| 1-myristoyl-2-arachidonoyl-GPC (14:0/20:4)* |  | [HMDB07883](http://www.hmdb.ca/metabolites/HMDB07883) | 1.00 | **0.53** | **0.53** | Lipid | Phosphatidylcholine (PC) |
| 1-palmitoyl-2-palmitoleoyl-GPC (16:0/16:1)* |  | [HMDB07969](http://www.hmdb.ca/metabolites/HMDB07969) | **1.72** | **1.38** | **0.81** | Lipid | Phosphatidylcholine (PC) |
| 1-palmitoyl-2-stearoyl-GPC (16:0/18:0) |  | [HMDB07970](http://www.hmdb.ca/metabolites/HMDB07970) | **0.91** | **0.80** | **0.87** | Lipid | Phosphatidylcholine (PC) |
| 1-palmitoleoyl-2-linolenoyl-GPC (16:1/18:3)* |  | [HMDB08008](http://www.hmdb.ca/metabolites/HMDB08008) | 1.19 | **0.52** | **0.44** | Lipid | Phosphatidylcholine (PC) |
| 1-stearoyl-2-oleoyl-GPC (18:0/18:1) |  | [HMDB08038](http://www.hmdb.ca/metabolites/HMDB08038) | **1.19** | 1.00 | **0.84** | Lipid | Phosphatidylcholine (PC) |
| 1-stearoyl-2-linoleoyl-GPC (18:0/18:2)* |  | [HMDB08039](http://www.hmdb.ca/metabolites/HMDB08039) | **0.84** | **0.77** | **0.92** | Lipid | Phosphatidylcholine (PC) |
| 1-oleoyl-2-docosahexaenoyl-GPC (18:1/22:6)* |  | [HMDB08123](http://www.hmdb.ca/metabolites/HMDB08123) | **0.82** | **0.67** | **0.83** | Lipid | Phosphatidylcholine (PC) |
| 1,2-dilinoleoyl-GPC (18:2/18:2) |  | [HMDB08138](http://www.hmdb.ca/metabolites/HMDB08138) | **0.65** | **0.38** | **0.58** | Lipid | Phosphatidylcholine (PC) |
| 1-linoleoyl-2-linolenoyl-GPC (18:2/18:3)* |  | [HMDB08141](http://www.hmdb.ca/metabolites/HMDB08141) | **0.71** | **0.30** | **0.41** | Lipid | Phosphatidylcholine (PC) |
| 1-linoleoyl-2-arachidonoyl-GPC (18:2/20:4n6)* |  | [HMDB08147](http://www.hmdb.ca/metabolites/HMDB08147) | **0.72** | **0.47** | **0.65** | Lipid | Phosphatidylcholine (PC) |
| 1-palmitoyl-2-linoleoyl-GPE (16:0/18:2) |  | [HMDB05322](http://www.hmdb.ca/metabolites/HMDB05322) | **1.22** | **1.66** | **1.36** | Lipid | Phosphatidylethanolamine (PE) |
| 1-palmitoyl-2-arachidonoyl-GPE (16:0/20:4)* |  | [HMDB05323](http://www.hmdb.ca/metabolites/HMDB05323) | **1.30** | **1.59** | **1.22** | Lipid | Phosphatidylethanolamine (PE) |
| 1-palmitoyl-2-docosahexaenoyl-GPE (16:0/22:6)* |  | [HMDB05324](http://www.hmdb.ca/metabolites/HMDB05324) | 0.91 | 1.14 | **1.24** | Lipid | Phosphatidylethanolamine (PE) |
| 1-stearoyl-2-docosahexaenoyl-GPE (18:0/22:6)* |  | [HMDB05334](http://www.hmdb.ca/metabolites/HMDB05334) | **0.79** | 0.99 | **1.25** | Lipid | Phosphatidylethanolamine (PE) |
| 1-stearoyl-2-oleoyl-GPG (18:0/18:1) |  |  | **0.56** | **0.43** | **0.77** | Lipid | Phosphatidylglycerol (PG) |
| 1-stearoyl-2-arachidonoyl-GPI (18:0/20:4) |  | [HMDB09815](http://www.hmdb.ca/metabolites/HMDB09815) | 0.97 | **0.73** | **0.75** | Lipid | Phosphatidylinositol (PI) |
| 1-linoleoyl-GPA (18:2)* |  | [HMDB07856](http://www.hmdb.ca/metabolites/HMDB07856) | **0.44** | **0.34** | **0.76** | Lipid | Lysophospholipid |
| 1-palmitoyl-GPC (16:0) |  | [HMDB10382](http://www.hmdb.ca/metabolites/HMDB10382) | **0.59** | **0.39** | **0.66** | Lipid | Lysophospholipid |
| 2-palmitoyl-GPC (16:0)* |  | [HMDB61702](http://www.hmdb.ca/metabolites/HMDB61702) | **0.59** | **0.42** | **0.70** | Lipid | Lysophospholipid |
| 1-palmitoleoyl-GPC (16:1)* |  | [HMDB10383](http://www.hmdb.ca/metabolites/HMDB10383) | **0.77** | **0.41** | **0.54** | Lipid | Lysophospholipid |
| 1-stearoyl-GPC (18:0) |  | [HMDB10384](http://www.hmdb.ca/metabolites/HMDB10384) | **0.55** | **0.34** | **0.62** | Lipid | Lysophospholipid |
| 1-oleoyl-GPC (18:1) |  | [HMDB02815](http://www.hmdb.ca/metabolites/HMDB02815) | **0.74** | **0.46** | **0.62** | Lipid | Lysophospholipid |
| 1-linoleoyl-GPC (18:2) | [C04100](http://www.genome.jp/dbget-bin/www_bget?cpd+C04100) | [HMDB10386](http://www.hmdb.ca/metabolites/HMDB10386) | **0.66** | **0.39** | **0.59** | Lipid | Lysophospholipid |
| 1-linolenoyl-GPC (18:3)* |  | [HMDB10388](http://www.hmdb.ca/metabolites/HMDB10388) | **0.79** | **0.34** | **0.43** | Lipid | Lysophospholipid |
| 1-arachidonoyl-GPC (20:4n6)* | [C05208](http://www.genome.jp/dbget-bin/www_bget?cpd+C05208) | [HMDB10395](http://www.hmdb.ca/metabolites/HMDB10395) | **0.77** | **0.54** | **0.71** | Lipid | Lysophospholipid |
| 1-lignoceroyl-GPC (24:0) |  | [HMDB10405](http://www.hmdb.ca/metabolites/HMDB10405) | **0.61** | **0.45** | **0.74** | Lipid | Lysophospholipid |
| 1-palmitoyl-GPE (16:0) |  | [HMDB11503](http://www.hmdb.ca/metabolites/HMDB11503) | 0.94 | **0.65** | **0.69** | Lipid | Lysophospholipid |
| 1-stearoyl-GPE (18:0) |  | [HMDB11130](http://www.hmdb.ca/metabolites/HMDB11130) | **0.78** | **0.54** | **0.69** | Lipid | Lysophospholipid |
| 2-stearoyl-GPE (18:0)* |  | [HMDB11129](http://www.hmdb.ca/metabolites/HMDB11129) | **0.74** | **0.53** | **0.72** | Lipid | Lysophospholipid |
| 1-oleoyl-GPE (18:1) |  | [HMDB11506](http://www.hmdb.ca/metabolites/HMDB11506) | 0.97 | **0.58** | **0.60** | Lipid | Lysophospholipid |
| 1-linoleoyl-GPE (18:2)* |  | [HMDB11507](http://www.hmdb.ca/metabolites/HMDB11507) | 1.06 | **0.59** | **0.55** | Lipid | Lysophospholipid |
| 1-arachidonoyl-GPE (20:4n6)* |  | [HMDB11517](http://www.hmdb.ca/metabolites/HMDB11517) | 1.14 | **0.75** | **0.65** | Lipid | Lysophospholipid |
| 1-palmitoyl-GPG (16:0)* |  |  | **0.81** | **0.64** | **0.79** | Lipid | Lysophospholipid |
| 1-linoleoyl-GPG (18:2)* |  |  | 1.05 | **0.87** | **0.83** | Lipid | Lysophospholipid |
| 1-(1-enyl-palmitoyl)-2-oleoyl-GPE (P-16:0/18:1)* |  | [HMDB11342](http://www.hmdb.ca/metabolites/HMDB11342) | **0.74** | **0.55** | **0.74** | Lipid | Plasmalogen |
| 1-(1-enyl-palmitoyl)-2-linoleoyl-GPE (P-16:0/18:2)* |  | [HMDB11343](http://www.hmdb.ca/metabolites/HMDB11343) | **0.62** | **0.33** | **0.53** | Lipid | Plasmalogen |
| 1-(1-enyl-palmitoyl)-2-arachidonoyl-GPE (P-16:0/20:4)* |  | [HMDB11352](http://www.hmdb.ca/metabolites/HMDB11352) | **0.67** | **0.42** | **0.63** | Lipid | Plasmalogen |
| 1-(1-enyl-stearoyl)-2-oleoyl-GPE (P-18:0/18:1) |  | [HMDB11375](http://www.hmdb.ca/metabolites/HMDB11375) | **0.65** | **0.46** | **0.71** | Lipid | Plasmalogen |
| 1-(1-enyl-stearoyl)-2-linoleoyl-GPE (P-18:0/18:2)* |  | [HMDB11376](http://www.hmdb.ca/metabolites/HMDB11376) | **0.62** | **0.36** | **0.59** | Lipid | Plasmalogen |
| 1-(1-enyl-palmitoyl)-2-linoleoyl-GPC (P-16:0/18:2)* |  | [HMDB11211](http://www.hmdb.ca/metabolites/HMDB11211) | **0.71** | **0.55** | **0.78** | Lipid | Plasmalogen |
| 1-(1-enyl-stearoyl)-2-arachidonoyl-GPE (P-18:0/20:4)* |  | [HMDB05779](http://www.hmdb.ca/metabolites/HMDB05779) | **0.67** | **0.42** | **0.62** | Lipid | Plasmalogen |
| 1-(1-enyl-palmitoyl)-GPC (P-16:0)* |  | [HMDB10407](http://www.hmdb.ca/metabolites/HMDB10407) | **0.59** | **0.37** | **0.63** | Lipid | Lysoplasmalogen |
| 1-(1-enyl-palmitoyl)-GPE (P-16:0)* |  |  | **0.44** | **0.24** | **0.53** | Lipid | Lysoplasmalogen |
| 1-(1-enyl-oleoyl)-GPE (P-18:1)* |  |  | **0.43** | **0.28** | **0.66** | Lipid | Lysoplasmalogen |
| 1-(1-enyl-stearoyl)-GPE (P-18:0)* |  |  | **0.46** | **0.24** | **0.51** | Lipid | Lysoplasmalogen |
| 1-palmitoleoylglycerol (16:1)* |  | [HMDB11565](http://www.hmdb.ca/metabolites/HMDB11565) | **1.90** | 0.97 | **0.51** | Lipid | Monoacylglycerol |
| 1-oleoylglycerol (18:1) |  | [HMDB11567](http://www.hmdb.ca/metabolites/HMDB11567) | 1.15 | **0.67** | **0.58** | Lipid | Monoacylglycerol |
| 1-linoleoylglycerol (18:2) |  |  | 1.41 | **0.77** | **0.54** | Lipid | Monoacylglycerol |
| 1-dihomo-linolenylglycerol (20:3) |  |  | 1.44 | **0.56** | **0.39** | Lipid | Monoacylglycerol |
| 1-arachidonylglycerol (20:4) | [C13857](http://www.genome.jp/dbget-bin/www_bget?cpd+C13857) | [HMDB11549](http://www.hmdb.ca/metabolites/HMDB11549) | 1.22 | **0.77** | **0.63** | Lipid | Monoacylglycerol |
| 1-docosahexaenoylglycerol (22:6) |  | [HMDB11587](http://www.hmdb.ca/metabolites/HMDB11587) | 0.97 | **0.36** | **0.37** | Lipid | Monoacylglycerol |
| 2-oleoylglycerol (18:1) |  | [HMDB11537](http://www.hmdb.ca/metabolites/HMDB11537) | 1.17 | **0.64** | **0.55** | Lipid | Monoacylglycerol |
| 2-linoleoylglycerol (18:2) |  | [HMDB11538](http://www.hmdb.ca/metabolites/HMDB11538) | **1.55** | **0.76** | **0.49** | Lipid | Monoacylglycerol |
| N-stearoyl-sphingosine (d18:1/18:0)* |  | [HMDB04950](http://www.hmdb.ca/metabolites/HMDB04950) | 1.18 | **2.32** | **1.96** | Lipid | Ceramides |
| ceramide (d18:1/20:0, d16:1/22:0, d20:1/18:0)* |  |  | **0.65** | 0.93 | **1.44** | Lipid | Ceramides |
| sphingomyelin (d18:0/18:0, d19:0/17:0)* |  | [HMDB12087](http://www.hmdb.ca/metabolites/HMDB12087) | 0.93 | **1.45** | **1.56** | Lipid | Dihydrosphingomyelins |
| hydroxypalmitoyl sphingomyelin (d18:1/16:0(OH))** |  |  | 0.94 | **1.12** | **1.19** | Lipid | Sphingomyelins |
| stearoyl sphingomyelin (d18:1/18:0) | [C00550](http://www.genome.jp/dbget-bin/www_bget?cpd+C00550) | [HMDB01348](http://www.hmdb.ca/metabolites/HMDB01348) | 0.93 | 1.12 | **1.21** | Lipid | Sphingomyelins |
| sphingomyelin (d17:1/14:0, d16:1/15:0)* |  |  | **0.62** | **0.44** | **0.70** | Lipid | Sphingomyelins |
| sphingomyelin (d18:2/14:0, d18:1/14:1)* |  |  | **0.85** | **0.67** | **0.78** | Lipid | Sphingomyelins |
| sphingomyelin (d18:1/20:2, d18:2/20:1, d16:1/22:2)* |  |  | 1.01 | **1.36** | **1.35** | Lipid | Sphingomyelins |
| sphingosine 1-phosphate | [C06124](http://www.genome.jp/dbget-bin/www_bget?cpd+C06124) | [HMDB00277](http://www.hmdb.ca/metabolites/HMDB00277) | 0.95 | **0.83** | **0.87** | Lipid | Sphingosines |
| 7-alpha-hydroxy-3-oxo-4-cholestenoate (7-Hoca) | [C17337](http://www.genome.jp/dbget-bin/www_bget?cpd+C17337) | [HMDB12458](http://www.hmdb.ca/metabolites/HMDB12458) | 0.96 | **0.77** | **0.80** | Lipid | Sterol |
| 3beta-hydroxy-5-cholestenoate | [C17333](http://www.genome.jp/dbget-bin/www_bget?cpd+C17333) |  | 0.95 | **0.72** | **0.76** | Lipid | Sterol |
| pregnenolone sulfate |  | [HMDB00774](http://www.hmdb.ca/metabolites/HMDB00774) | **0.76** | 1.28 | **1.69** | Lipid | Pregnenolone Steroids |
| 21-hydroxypregnenolone disulfate |  |  | **0.79** | **1.53** | **1.94** | Lipid | Pregnenolone Steroids |
| pregnenediol sulfate (C21H34O5S)* |  |  | **0.71** | 1.36 | **1.91** | Lipid | Pregnenolone Steroids |
| pregnenediol disulfate (C21H34O8S2)* |  |  | 0.87 | **1.93** | **2.22** | Lipid | Pregnenolone Steroids |
| pregnenetriol sulfate* |  |  | **0.66** | 1.51 | **2.30** | Lipid | Pregnenolone Steroids |
| pregnenetriol disulfate* |  |  | **0.71** | **2.13** | **3.00** | Lipid | Pregnenolone Steroids |
| 5alpha-pregnan-3beta,20beta-diol monosulfate (1) |  |  | 0.83 | 1.14 | **1.36** | Lipid | Progestin Steroids |
| 5alpha-pregnan-3beta,20alpha-diol disulfate |  |  | 0.96 | **1.39** | **1.45** | Lipid | Progestin Steroids |
| 5alpha-pregnan-diol disulfate |  |  | 1.54 | **3.63** | **2.35** | Lipid | Progestin Steroids |
| pregnanediol-3-glucuronide |  | [HMDB10318](http://www.hmdb.ca/metabolites/HMDB10318) | 0.96 | **1.64** | **1.71** | Lipid | Progestin Steroids |
| pregnanolone/allopregnanolone sulfate |  |  | 0.98 | **1.44** | **1.48** | Lipid | Progestin Steroids |
| cortisol | [C00735](http://www.genome.jp/dbget-bin/www_bget?cpd+C00735) | [HMDB00063](http://www.hmdb.ca/metabolites/HMDB00063) | **0.70** | 1.09 | **1.56** | Lipid | Corticosteroids |
| cortolone glucuronide (1) |  |  | **0.96** | 1.68 | **1.75** | Lipid | Corticosteroids |
| tetrahydrocortisol sulfate (1) |  |  | 1.19 | **6.33** | **5.31** | Lipid | Corticosteroids |
| 5alpha-androstan-3alpha,17alpha-diol disulfate |  |  | 1.26 | **2.13** | **1.69** | Lipid | Androgenic Steroids |
| androstenediol (3beta,17beta) monosulfate (1) |  | [HMDB03818](http://www.hmdb.ca/metabolites/HMDB03818) | **0.65** | 1.10 | **1.69** | Lipid | Androgenic Steroids |
| androstenediol (3beta,17beta) disulfate (1) | [C04295](http://www.genome.jp/dbget-bin/www_bget?cpd+C04295) | [HMDB03818](http://www.hmdb.ca/metabolites/HMDB03818) | **0.60** | 0.85 | **1.41** | Lipid | Androgenic Steroids |
| androstenediol (3beta,17beta) disulfate (2) | [C04295](http://www.genome.jp/dbget-bin/www_bget?cpd+C04295) | [HMDB03818](http://www.hmdb.ca/metabolites/HMDB03818) | 0.86 | 1.15 | **1.34** | Lipid | Androgenic Steroids |
| androstenediol (3alpha, 17alpha) monosulfate (3) |  |  | 0.81 | **1.15** | **1.41** | Lipid | Androgenic Steroids |
| 11beta-hydroxyandrosterone glucuronide |  |  | **0.69** | 1.25 | **1.82** | Lipid | Androgenic Steroids |
| estrone 3-sulfate | [C02538](http://www.genome.jp/dbget-bin/www_bget?cpd+C02538) | [HMDB01425](http://www.hmdb.ca/metabolites/HMDB01425) | 0.94 | **1.86** | **1.99** | Lipid | Estrogenic Steroids |
| glycochenodeoxycholate | [C05466](http://www.genome.jp/dbget-bin/www_bget?cpd+C05466) | [HMDB00637](http://www.hmdb.ca/metabolites/HMDB00637) | 1.21 | **0.80** | **0.66** | Lipid | Primary Bile Acid Metabolism |
| glyco-beta-muricholate** |  |  | 0.81 | **0.51** | **0.63** | Lipid | Primary Bile Acid Metabolism |
| glycodeoxycholate | [C05464](http://www.genome.jp/dbget-bin/www_bget?cpd+C05464) | [HMDB00631](http://www.hmdb.ca/metabolites/HMDB00631) | 1.02 | **0.68** | **0.66** | Lipid | Secondary Bile Acid Metabolism |
| ursodeoxycholate | [C07880](http://www.genome.jp/dbget-bin/www_bget?cpd+C07880) | [HMDB00946](http://www.hmdb.ca/metabolites/HMDB00946) | **0.70** | **0.28** | **0.40** | Lipid | Secondary Bile Acid Metabolism |
| isoursodeoxycholate | [C17662](http://www.genome.jp/dbget-bin/www_bget?cpd+C17662) | [HMDB00686](http://www.hmdb.ca/metabolites/HMDB00686) | 0.99 | **0.31** | **0.31** | Lipid | Secondary Bile Acid Metabolism |
| glycoursodeoxycholate |  | [HMDB00708](http://www.hmdb.ca/metabolites/HMDB00708) | 2.40 | **0.59** | **0.25** | Lipid | Secondary Bile Acid Metabolism |
| glycohyocholate |  |  | 0.90 | **0.86** | **0.96** | Lipid | Secondary Bile Acid Metabolism |
| glycocholenate sulfate* |  |  | 1.01 | **1.47** | **1.45** | Lipid | Secondary Bile Acid Metabolism |
| taurocholenate sulfate* |  |  | 1.16 | **2.89** | **2.48** | Lipid | Secondary Bile Acid Metabolism |
| 3b-hydroxy-5-cholenoic acid |  | [HMDB00308](http://www.hmdb.ca/metabolites/HMDB00308) | **0.71** | **0.47** | **0.66** | Lipid | Secondary Bile Acid Metabolism |
| urate | [C00366](http://www.genome.jp/dbget-bin/www_bget?cpd+C00366) | [HMDB00289](http://www.hmdb.ca/metabolites/HMDB00289) | 1.04 | **0.95** | **0.91** | Nucleotide | Purine Metabolism, (Hypo)Xanthine/Inosine containing |
| allantoin | [C02350](http://www.genome.jp/dbget-bin/www_bget?cpd+C02350) | [HMDB00462](http://www.hmdb.ca/metabolites/HMDB00462) | 1.05 | **0.79** | **0.76** | Nucleotide | Purine Metabolism, (Hypo)Xanthine/Inosine containing |
| adenosine 5'-diphosphate (ADP) | [C00008](http://www.genome.jp/dbget-bin/www_bget?cpd+C00008) | [HMDB01341](http://www.hmdb.ca/metabolites/HMDB01341) | **3.29** | **2.40** | **0.73** | Nucleotide | Purine Metabolism, Adenine containing |
| N1-methyladenosine | [C02494](http://www.genome.jp/dbget-bin/www_bget?cpd+C02494) | [HMDB03331](http://www.hmdb.ca/metabolites/HMDB03331) | 1.03 | **1.28** | **1.24** | Nucleotide | Purine Metabolism, Adenine containing |
| N6-carbamoylthreonyladenosine |  | [HMDB41623](http://www.hmdb.ca/metabolites/HMDB41623) | **1.50** | **1.74** | **1.17** | Nucleotide | Purine Metabolism, Adenine containing |
| N2,N2-dimethylguanosine |  | [HMDB04824](http://www.hmdb.ca/metabolites/HMDB04824) | **1.25** | **1.50** | **1.19** | Nucleotide | Purine Metabolism, Guanine containing |
| uridine | [C00299](http://www.genome.jp/dbget-bin/www_bget?cpd+C00299) | [HMDB00296](http://www.hmdb.ca/metabolites/HMDB00296) | **0.88** | **0.74** | **0.85** | Nucleotide | Pyrimidine Metabolism, Uracil containing |
| 2'-O-methyluridine |  |  | **0.74** | **0.90** | **1.23** | Nucleotide | Pyrimidine Metabolism, Uracil containing |
| 5,6-dihydrouracil | [C00429](http://www.genome.jp/dbget-bin/www_bget?cpd+C00429) | [HMDB00076](http://www.hmdb.ca/metabolites/HMDB00076) | **0.88** | **0.77** | **0.87** | Nucleotide | Pyrimidine Metabolism, Uracil containing |
| 3-methylcytidine |  |  | **1.23** | **1.44** | **1.17** | Nucleotide | Pyrimidine Metabolism, Cytidine containing |
| 3-aminoisobutyrate | [C05145](http://www.genome.jp/dbget-bin/www_bget?cpd+C05145) | [HMDB03911](http://www.hmdb.ca/metabolites/HMDB03911) | 1.21 | **1.53** | **1.26** | Nucleotide | Pyrimidine Metabolism, Thymine containing |
| nicotinamide | [C00153](http://www.genome.jp/dbget-bin/www_bget?cpd+C00153) | [HMDB01406](http://www.hmdb.ca/metabolites/HMDB01406) | **0.77** | **0.61** | **0.79** | Cofactors and Vitamins | Nicotinate and Nicotinamide Metabolism |
| nicotinamide riboside | [C03150](http://www.genome.jp/dbget-bin/www_bget?cpd+C03150) | [HMDB00855](http://www.hmdb.ca/metabolites/HMDB00855) | **0.64** | 1.12 | **1.77** | Cofactors and Vitamins | Nicotinate and Nicotinamide Metabolism |
| 1-methylnicotinamide | [C02918](http://www.genome.jp/dbget-bin/www_bget?cpd+C02918) | [HMDB00699](http://www.hmdb.ca/metabolites/HMDB00699) | **0.59** | **0.57** | **0.97** | Cofactors and Vitamins | Nicotinate and Nicotinamide Metabolism |
| trigonelline (N'-methylnicotinate) | [C01004](http://www.genome.jp/dbget-bin/www_bget?cpd+C01004) | [HMDB00875](http://www.hmdb.ca/metabolites/HMDB00875) | 0.95 | **0.70** | **0.74** | Cofactors and Vitamins | Nicotinate and Nicotinamide Metabolism |
| N1-Methyl-2-pyridone-5-carboxamide | [C05842](http://www.genome.jp/dbget-bin/www_bget?cpd+C05842) | [HMDB04193](http://www.hmdb.ca/metabolites/HMDB04193) | 1.09 | **0.94** | **0.86** | Cofactors and Vitamins | Nicotinate and Nicotinamide Metabolism |
| N1-Methyl-4-pyridone-3-carboxamide | [C05843](http://www.genome.jp/dbget-bin/www_bget?cpd+C05843) | [HMDB04194](http://www.hmdb.ca/metabolites/HMDB04194) | 1.19 | **1.01** | **0.84** | Cofactors and Vitamins | Nicotinate and Nicotinamide Metabolism |
| ascorbic acid 2-sulfate |  |  | 1.39 | 1.02 | **0.74** | Cofactors and Vitamins | Ascorbate and Aldarate Metabolism |
| threonate | [C01620](http://www.genome.jp/dbget-bin/www_bget?cpd+C01620) | [HMDB00943](http://www.hmdb.ca/metabolites/HMDB00943) | **0.60** | **0.61** | **1.02** | Cofactors and Vitamins | Ascorbate and Aldarate Metabolism |
| oxalate (ethanedioate) | [C00209](http://www.genome.jp/dbget-bin/www_bget?cpd+C00209) | [HMDB02329](http://www.hmdb.ca/metabolites/HMDB02329) | **0.63** | **0.54** | **0.86** | Cofactors and Vitamins | Ascorbate and Aldarate Metabolism |
| gamma-CEHC |  | [HMDB01931](http://www.hmdb.ca/metabolites/HMDB01931) | **0.62** | **0.35** | **0.57** | Cofactors and Vitamins | Tocopherol Metabolism |
| gamma-CEHC glucuronide* |  |  | 0.98 | **0.72** | **0.73** | Cofactors and Vitamins | Tocopherol Metabolism |
| delta-CEHC |  |  | 0.85 | **0.40** | **0.47** | Cofactors and Vitamins | Tocopherol Metabolism |
| delta-CEHC glucuronide |  |  | 1.22 | **0.81** | **0.66** | Cofactors and Vitamins | Tocopherol Metabolism |
| retinol (Vitamin A) | [C00473](http://www.genome.jp/dbget-bin/www_bget?cpd+C00473) | [HMDB00305](http://www.hmdb.ca/metabolites/HMDB00305) | **0.76** | **0.53** | **0.69** | Cofactors and Vitamins | Vitamin A Metabolism |
| carotene diol (1) |  |  | **0.69** | **0.54** | **0.79** | Cofactors and Vitamins | Vitamin A Metabolism |
| carotene diol (2) |  |  | **0.79** | **0.59** | **0.75** | Cofactors and Vitamins | Vitamin A Metabolism |
| pyridoxate | [C00847](http://www.genome.jp/dbget-bin/www_bget?cpd+C00847) | [HMDB00017](http://www.hmdb.ca/metabolites/HMDB00017) | **0.42** | 0.59 | **1.42** | Cofactors and Vitamins | Vitamin B6 Metabolism |
| catechol sulfate |  | [HMDB59724](http://www.hmdb.ca/metabolites/HMDB59724) | **0.57** | **0.41** | **0.73** | Xenobiotics | Benzoate Metabolism |
| guaiacol sulfate |  | [HMDB60013](http://www.hmdb.ca/metabolites/HMDB60013) | **0.60** | **0.64** | **1.07** | Xenobiotics | Benzoate Metabolism |
| 4-allylcatechol sulfate |  |  | **0.49** | **0.15** | **0.30** | Xenobiotics | Benzoate Metabolism |
| 4-acetylphenol sulfate |  |  | 0.81 | 0.24 | **0.29** | Xenobiotics | Benzoate Metabolism |
| 4-ethylphenylsulfate | [C13637](http://www.genome.jp/dbget-bin/www_bget?cpd+C13637) | [HMDB62551](http://www.hmdb.ca/metabolites/HMDB62551) | 0.95 | **0.33** | **0.35** | Xenobiotics | Benzoate Metabolism |
| 4-vinylphenol sulfate | [C05627](http://www.genome.jp/dbget-bin/www_bget?cpd+C05627) | [HMDB62775](http://www.hmdb.ca/metabolites/HMDB62775) | **0.57** | **0.30** | **0.52** | Xenobiotics | Benzoate Metabolism |
| propyl 4-hydroxybenzoate | [D01422](http://www.genome.jp/dbget-bin/www_bget?cpd+D01422) | [HMDB32574](http://www.hmdb.ca/metabolites/HMDB32574) | 1.07 | **4.53** | **4.23** | Xenobiotics | Benzoate Metabolism |
| cotinine |  | [HMDB01046](http://www.hmdb.ca/metabolites/HMDB01046) | **0.11** | 0.44 | **4.07** | Xenobiotics | Tobacco Metabolite |
| gluconate | [C00257](http://www.genome.jp/dbget-bin/www_bget?cpd+C00257) | [HMDB00625](http://www.hmdb.ca/metabolites/HMDB00625) | 9.65 | **37.08** | **3.84** | Xenobiotics | Food Component/Plant |
| 4-acetylcatechol sulfate (1) |  |  | **0.58** | **0.45** | **0.77** | Xenobiotics | Food Component/Plant |
| genistein sulfate* |  |  | **0.33** | **0.03** | **0.10** | Xenobiotics | Food Component/Plant |
| homostachydrine* | [C08283](http://www.genome.jp/dbget-bin/www_bget?cpd+C08283) | [HMDB33433](http://www.hmdb.ca/metabolites/HMDB33433) | 1.15 | **1.31** | **1.14** | Xenobiotics | Food Component/Plant |
| mannonate* |  |  | 1.70 | **3.68** | **2.16** | Xenobiotics | Food Component/Plant |
| piperine | [C03882](http://www.genome.jp/dbget-bin/www_bget?cpd+C03882) | [HMDB29377](http://www.hmdb.ca/metabolites/HMDB29377) | **0.38** | **0.17** | **0.46** | Xenobiotics | Food Component/Plant |
| glucuronide of piperine metabolite C17H21NO3 (4)* |  |  | 0.57 | **0.24** | **0.42** | Xenobiotics | Food Component/Plant |
| sulfate of piperine metabolite C16H19NO3 (2)* |  |  | **0.47** | **0.20** | **0.43** | Xenobiotics | Food Component/Plant |
| sulfate of piperine metabolite C16H19NO3 (3)* |  |  | **0.47** | **0.21** | **0.45** | Xenobiotics | Food Component/Plant |
| sulfate of piperine metabolite C18H21NO3 (1)* |  |  | **0.45** | **0.22** | **0.49** | Xenobiotics | Food Component/Plant |
| sulfate of piperine metabolite C18H21NO3 (3)* |  |  | **0.46** | **0.24** | **0.52** | Xenobiotics | Food Component/Plant |
| saccharin | [D01085](http://www.genome.jp/dbget-bin/www_bget?cpd+D01085) | [HMDB29723](http://www.hmdb.ca/metabolites/HMDB29723) | 0.99 | 0.80 | **0.81** | Xenobiotics | Food Component/Plant |
| 4-allylphenol sulfate |  |  | **0.81** | **0.47** | **0.58** | Xenobiotics | Food Component/Plant |
| methyl glucopyranoside (alpha + beta) |  |  | **0.42** | **0.33** | **0.79** | Xenobiotics | Food Component/Plant |
| daidzein sulfate (2) |  |  | **0.37** | **0.09** | **0.25** | Xenobiotics | Food Component/Plant |
| 3,4-methyleneheptanoate |  |  | 1.04 | **0.57** | **0.55** | Xenobiotics | Food Component/Plant |
| 4-acetamidophenol | [C06804](http://www.genome.jp/dbget-bin/www_bget?cpd+C06804) | [HMDB01859](http://www.hmdb.ca/metabolites/HMDB01859) | 0.13 | 5.27 | **41.18** | Xenobiotics | Drug - Analgesics, Anesthetics |
| 3-(N-acetyl-L-cystein-S-yl) acetaminophen |  |  | 0.98 | **7.26** | **7.37** | Xenobiotics | Drug - Analgesics, Anesthetics |
| 4-acetamidophenylglucuronide |  | [HMDB10316](http://www.hmdb.ca/metabolites/HMDB10316) | 0.95 | **25.92** | **27.19** | Xenobiotics | Drug - Analgesics, Anesthetics |
| tramadol | [C07153](http://www.genome.jp/dbget-bin/www_bget?cpd+C07153) | [HMDB14339](http://www.hmdb.ca/metabolites/HMDB14339) | 0.60 | **14.28** | **23.92** | Xenobiotics | Drug - Analgesics, Anesthetics |
| O-desmethyltramadol |  | [HMDB60997](http://www.hmdb.ca/metabolites/HMDB60997) | 1.00 | **1.51** | **1.52** | Xenobiotics | Drug - Analgesics, Anesthetics |
| O-desmethyltramadol glucuronide |  |  | 0.80 | **2.46** | **3.08** | Xenobiotics | Drug - Analgesics, Anesthetics |
| N-desmethyl tramadol |  | [HMDB61007](http://www.hmdb.ca/metabolites/HMDB61007) | 1.00 | **1.69** | **1.69** | Xenobiotics | Drug - Analgesics, Anesthetics |
| metoprolol | [D02358](http://www.genome.jp/dbget-bin/www_bget?cpd+D02358) | [HMDB01932](http://www.hmdb.ca/metabolites/HMDB01932) | **4.45** | **12.37** | **2.78** | Xenobiotics | Drug - Cardiovascular |
| metoprolol acid metabolite* |  |  | **7.75** | **22.36** | **2.88** | Xenobiotics | Drug - Cardiovascular |
| alpha-hydroxymetoprolol |  | [HMDB60994](http://www.hmdb.ca/metabolites/HMDB60994) | **2.88** | **11.40** | **3.96** | Xenobiotics | Drug - Cardiovascular |
| atenolol | [D00235](http://www.genome.jp/dbget-bin/www_bget?cpd+D00235) | [HMDB01924](http://www.hmdb.ca/metabolites/HMDB01924) | **10.55** | **13.39** | **1.27** | Xenobiotics | Drug - Cardiovascular |
| nifedipine | D00437   C07266 |  | **2.92** | **11.58** | **3.97** | Xenobiotics | Drug - Cardiovascular |
| diltiazem | [C06958](http://www.genome.jp/dbget-bin/www_bget?cpd+C06958) | [HMDB14487](http://www.hmdb.ca/metabolites/HMDB14487) | 1.00 | **187.38** | **187.38** | Xenobiotics | Drug - Cardiovascular |
| methoxamine | [C07513](http://www.genome.jp/dbget-bin/www_bget?cpd+C07513) | [HMDB14861](http://www.hmdb.ca/metabolites/HMDB14861) | 5.18 | **52.56** | **10.14** | Xenobiotics | Drug - Cardiovascular |
| pantoprazole | [C11806](http://www.genome.jp/dbget-bin/www_bget?cpd+C11806) | [HMDB05017](http://www.hmdb.ca/metabolites/HMDB05017) | 1.00 | **94.09** | **94.09** | Xenobiotics | Drug - Gastrointestinal |
| diphenhydramine | [C06960](http://www.genome.jp/dbget-bin/www_bget?cpd+C06960) | [HMDB01927](http://www.hmdb.ca/metabolites/HMDB01927) | 0.86 | **36.89** | **42.69** | Xenobiotics | Drug - Respiratory |
| 2,6-dihydroxybenzoic acid |  | [HMDB13676](http://www.hmdb.ca/metabolites/HMDB13676) | 0.78 | **0.34** | **0.43** | Xenobiotics | Drug - Topical Agents |
| 3-acetylphenol sulfate |  |  | **2.18** | **1.72** | **0.79** | Xenobiotics | Chemical |
| 2-aminophenol sulfate |  | [HMDB61116](http://www.hmdb.ca/metabolites/HMDB61116) | 0.75 | **0.39** | **0.52** | Xenobiotics | Chemical |
| methylnaphthyl sulfate (2)* |  |  | **0.34** | 0.73 | **2.17** | Xenobiotics | Chemical |
| trizma acetate | [C07182](http://www.genome.jp/dbget-bin/www_bget?cpd+C07182) |  | **5.45** | **40.72** | **7.47** | Xenobiotics | Chemical |
| glucuronide of C10H18O2 (1)* |  |  | **10.46** | **6.43** | **0.62** | Partially Characterized Molecules | Partially Characterized Molecules |
| glucuronide of C12H22O4 (1)* |  |  | **5.75** | 1.01 | **0.18** | Partially Characterized Molecules | Partially Characterized Molecules |
| glycine conjugate of C10H14O2 (1)* |  |  | 0.73 | **0.64** | **0.88** | Partially Characterized Molecules | Partially Characterized Molecules |
| branched-chain, straight-chain, or cyclopropyl 10:1 fatty acid (1)* |  |  | 0.78 | **0.49** | **0.62** | Partially Characterized Molecules | Partially Characterized Molecules |
| branched-chain, straight-chain, or cyclopropyl 12:1 fatty acid* |  |  | **0.54** | **0.44** | **0.81** | Partially Characterized Molecules | Partially Characterized Molecules |
| GlcNAc sulfate conjugate of C21H34O2 steroid** |  |  | 1.49 | **4.39** | **2.96** | Partially Characterized Molecules | Partially Characterized Molecules |

Red and green shaded cells indicate p≤0.05 (red indicates that the mean values are significantly higher for that comparison; green values significantly lower.

**Table S4. Characteristics of the patients included in the validation cohort**

| **Demographic Information** | **TAA (n = 183)** | **TAD (n = 86)** | ***P* value** |
| --- | --- | --- | --- |
| Male (%) | 143(78.1%) | 54(62.8%) | 0.0080 |
| Age, median (IQR), year-old | 46.5(35-55) | 46(35-55) | 0.0582 |
| Hypertension (%) | 21(11.4%) | 32(37.2%) | <0.0001 |
| Hyperlipidemia (%) | 15(8.2%) | 20 (23.3%) | 0.0006 |
| Diabetes (%) | 2(1.1%) | 1(1.2%) | 0.9563 |

The differences in gender ratio, and the proportion of hypertension, hyperlipidemia and diabetes between patients with TAA and TAD were evaluated by the Chi-square test. Besides, the difference of age between patients with TAA and TAD was evaluated by the t-test.
